# Diversity and distribution of marine heterotrophic bacteria from a large culture collection

**DOI:** 10.1186/s12866-020-01884-7

**Published:** 2020-07-13

**Authors:** Isabel Sanz-Sáez, Guillem Salazar, Pablo Sánchez, Elena Lara, Marta Royo-Llonch, Elisabet L. Sà, Teresa Lucena, María J. Pujalte, Dolors Vaqué, Carlos M. Duarte, Josep M. Gasol, Carlos Pedrós-Alió, Olga Sánchez, Silvia G. Acinas

**Affiliations:** 1 Department of Marine Biology and Oceanography, Institut de Ciències del Mar (CSIC), 08003 Barcelona, Spain; 2 Department of Biology, Institute of Microbiology, ETH Zurich, Vladimir-Prelog-Weg 1-5/10, CH-8093 Zurich, Switzerland; 3 Institute of Marine Sciences (CNR-ISMAR), National Research Council, Castello 2737/F Arsenale-Tesa 104, 30122 Venezia, Italy; 4 Departamento de Microbiología y Ecología and Colección Española de Cultivos Tipo (CECT), Universitat de València, Valencia, Spain; 5 Red Sea Research Center, King Abdullah University of Science and Technology (KAUST), Thuwal, 23955-6900 Saudi Arabia; 6 Computational Bioscience Research Center (CBRC), King Abdullah University of Science and Technology (KAUST), Thuwal, 23955-6900 Saudi Arabia; 7 Department of Systems Biology, Centro Nacional de Biotecnología (CNB), CSIC, Madrid, Spain; 8 Departament de Genètica i Microbiologia, Facultat de Biociències, Universitat Autònoma de Barcelona, 08193 Bellaterra, Spain

**Keywords:** Bacterial isolates, Deep ocean, Photic ocean, Diversity

## Abstract

**Background:**

Isolation of marine microorganisms is fundamental to gather information about their physiology, ecology and genomic content. To date, most of the bacterial isolation efforts have focused on the photic ocean leaving the deep ocean less explored. We have created a marine culture collection of heterotrophic bacteria (MARINHET) using a standard marine medium comprising a total of 1561 bacterial strains, and covering a variety of oceanographic regions from different seasons and years, from 2009 to 2015. Specifically, our marine collection contains isolates from both photic (817) and aphotic layers (744), including the mesopelagic (362) and the bathypelagic (382), from the North Western Mediterranean Sea, the North and South Atlantic Ocean, the Indian, the Pacific, and the Arctic Oceans. We described the taxonomy, the phylogenetic diversity and the biogeography of a fraction of the marine culturable microorganisms to enhance our knowledge about which heterotrophic marine isolates are recurrently retrieved across oceans and along different depths.

**Results:**

The partial sequencing of the 16S rRNA gene of all isolates revealed that they mainly affiliate with the classes *Alphaproteobacteria* (35.9%), *Gammaproteobacteria* (38.6%), and phylum *Bacteroidetes* (16.5%). In addition, *Alteromonas* and *Erythrobacter* genera were found the most common heterotrophic bacteria in the ocean growing in solid agar medium. When comparing all photic, mesopelagic, and bathypelagic isolates sequences retrieved from different stations, 37% of them were 100% identical. This percentage increased up to 59% when mesopelagic and bathypelagic strains were grouped as the aphotic dataset and compared to the photic dataset of isolates, indicating the ubiquity of some bacterial isolates along different ocean depths. Finally, we isolated three strains that represent a new species, and the genome comparison and phenotypic characterization of two of these strains (ISS653 and ISS1889) concluded that they belong to a new species within the genus *Mesonia*.

**Conclusions:**

Overall, this study highlights the relevance of culture-dependent studies, with focus on marine isolated bacteria from different oceanographic regions and depths, to provide a more comprehensive view of the culturable marine bacteria as part of the total marine microbial diversity.

## Background

Traditional culturing methods allow the isolation of microorganisms from natural samples with the possibility to sequence their genome, perform physiological/experimental assays and, thus, infer their functional and ecological role in detail. Moreover, microbial cultures can retrieve diversity usually not recovered by molecular methodologies, particularly bacteria belonging to the “rare biosphere”, i.e. bacterial species that are present in very low abundances in the environment [1, 2]. The overlap between isolated microorganisms and those belonging to the uncultured majority is relatively low in molecular surveys, and efforts to culture bacteria from the ocean often yield isolates that do not have their corresponding 16S rRNA gene sequences deposited in sequence databases [3, 4]. As a consequence, isolation of microorganisms by culture-dependent techniques, and their comparison to data obtained from high-throughput sequencing techniques (HTS), remains a fundamental tool to fully understand the whole range of bacterioplankton diversity found in the ocean. In addition, isolation is so far a requisite for the description of new microbial species.

Most of the studies targeting the marine heterotrophic culturable bacteria have focused on the upper ocean (0–200 m depth) or on specific oceanographic regions [4–7], while studies covering different depths are less frequent [8–10]. Efforts to culture bacteria from the deep ocean (> 200 m) have focused mostly on isolates from hydrothermal vents [11–13], whale carcasses [14], trenches [15], and deep-sea sediments [10, 16–19]. Thus, very few studies have analyzed the diversity of isolates from mesopelagic (in particular from regions with oxygen minimum zone areas) [9, 20–22], the bathypelagic and abyssopelagic waters [8, 23–25], and those available were mainly done at a local or regional scale. Therefore, a study of the culturable microorganisms covering different layers including underexplored areas such as the mesopelagic and the bathypelagic areas is missing.

Here we present an extensive marine heterotrophic bacterial culture collection (MARINHET) with 1561 marine bacteria retrieved from different ocean depths from the Mediterranean Sea, the North and South Atlantic Oceans, the Indian, the Pacific, and the Arctic Oceans, covering diverse latitudes, from different seasons and years from 2009 to 2015. We used well established marine solid media (Zobell agar and Marine Agar 2216) in order to describe the fraction of the bacterioplankton community than can be commonly isolated under laboratory conditions (nutrient rich medium, standard oxygen concentrations and atmospheric pressure). Therefore, to the best of our knowledge, we have created the first extensive marine heterotrophic bacterial culture collection, including isolates from different depths and oceanographic regions, that were retrieved through a standard methodology. Analyses of the partial 16S rRNA gene sequences (average 526 bp, covering V3 to V5 regions), allowed us: (i) to identify the taxonomy of those isolates distributed along the water column, (ii) to explore the phylogenetic diversity and the potential differences between depths, (iii) to reveal the most common distributed heterotrophic culturable bacteria across oceans and depths, (iv) to describe the biogeography of the most abundant isolates recovered, (v) to compare the isolates 16S rRNA sequences with available HTS 16S rRNA sequences derived from samples of the same oceanographic expeditions, and (vi) to unveil some novel isolated bacterial strains.

## Results

### Taxonomic and phylogenetic diversity of the MARINHET culture collection

**Fig. 1:**
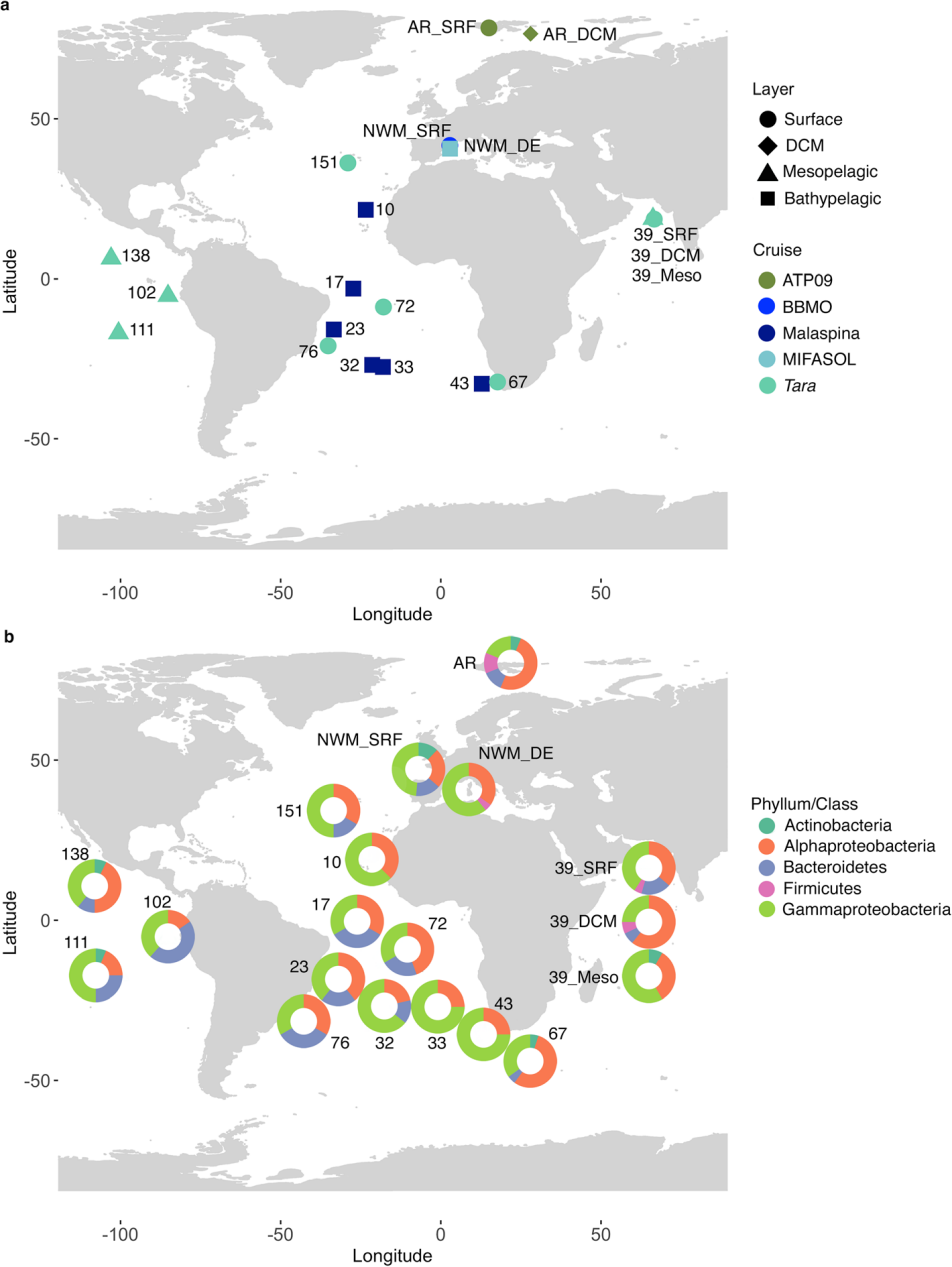
Map showing the sampling stations of the present study. **a** Position of the samples used for isolation. DCM: deep chlorophyll maximum. **b** Pie charts indicating the proportion of isolates retrieved affiliating with the different phyla, or classes in the case of Proteobacteria

A total of 1561 bacterial strains were isolated from 19 marine stations, eight photic-layer, four mesopelagic, and seven bathypelagic samples (Fig. 1a). The partial 16S rRNA sequences of the cultured strains were grouped into operational taxonomic units (isolated OTUs, referred hereafter as iOTUs) using 99% similarity thresholds. *Alphaproteobacteria* and *Gammaproteobacteria* iOTUs dominated in all stations (Fig. 1b). *Bacteroidetes* isolates were present in all photic stations, but were not retrieved in the Indian mesopelagic sample ST39 or in the Atlantic bathypelagic samples ST10, ST33 and ST43. *Actinobacteria* isolates were retrieved only from six stations including photic and mesopelagic but not from bathypelagic samples. Finally, *Firmicutes* could be only isolated from photic samples of the Arctic and Indian Ocean during the time of sampling (Fig. 1b).

**Fig. 2:**
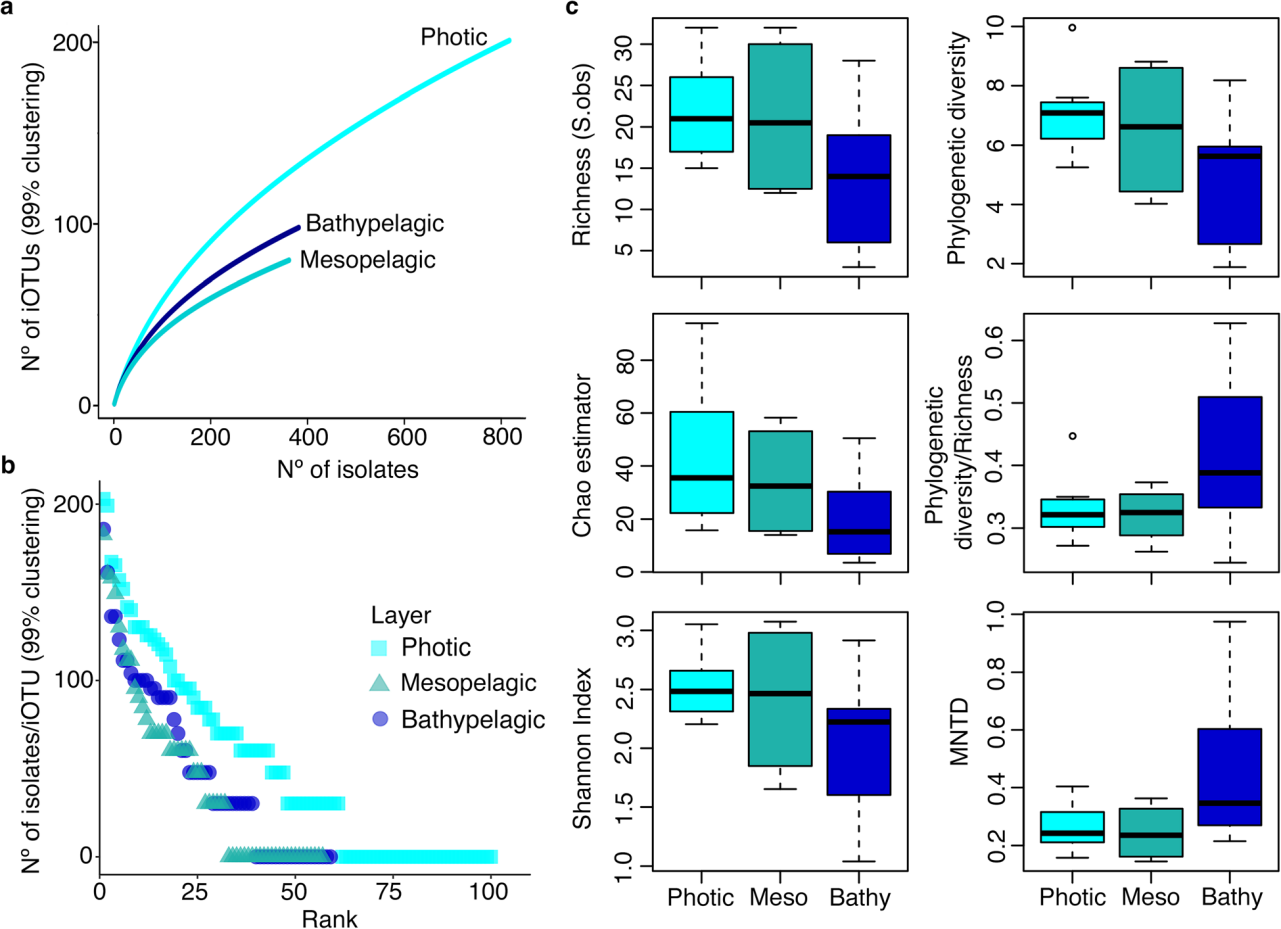
Diversity measures per layer studied. **a** Rarefaction curves extracted from the non-rarefied iOTU Table (99% clustering). **b** Rank abundance plots showing the number of isolates per iOTU (at 99% clustering) obtained in the three layers studied also for the non-rarefied iOTU Table. Y axis are in log10 scale. Photic: surface and deep chlorophyll maximum (DCM); Meso: mesopelagic; and Bathy: bathypelagic ocean. **c** Alpha-diversity measures using OTU-based (left panels) and phylogenetic (right panels) approaches. MNTD: mean nearest taxon distance.

If we group the different stations per depth, Good’s coverage analyses per layer, which is an estimator of the percentage of total species represented in a sample, ranged from 56.1 to 70.5% (Table 1). These results indicated that the isolates dataset, even if not saturated, represents a reasonable inventory of the culturable heterotrophic marine bacteria. The number of iOTUs detected was slightly higher in the photic layer for the non-rarefied iOTU table, but similar in all depths for the rarefied iOTU table, being the mesopelagic the layer with the lowest observed values (Table 1). Rarefaction curves showed also slightly higher richness for the photic samples compared to the mesopelagic and the bathypelagic, but they did not reach an asymptote (Fig. 2a and Supplementary Fig. S1a in Additional file 1). On the other hand, rank abundance plots of the non-rarefied (Fig. 2b) and rarefied iOTU tables (Supplementary Fig. S1b in Additional file 1) presented, for the three depths studied, a steep curve, which is indicative of low evenness. Thus, there were a few abundant iOTUs with a large number of representatives and a large proportion of iOTUs that had few representatives (rare iOTUs). Therefore, we also calculated the richness and diversity metrics of each depth using OTU-based and phylogenetic approaches. All three metrics of OTU-based alpha diversity used (Species observed (S.obs) or n° of iOTUs, Chao1 and Shannon indexes, Fig. 2c) decreased with depth but not significative differences were found between layers (ANOVA test: S.obs: *P*-value = 0.152; Chao richness estimator: P-value = 0.191; Shannon diversity index: P-value = 0.183). The three measures of phylogenetic diversity, Faith’s phylogenetic diversity (PD) [26], the PD divided by the number of iOTUs (PD/iOTUs), and the mean nearest taxon distance (MNTD) [27], were not significantly different between depths (ANOVA test: PD: P-value = 0.093; PD/iOTUs: P-value = 0.159; MNTD: P- value = 0.107), although a higher mean in phylogenetic diversity was observed in the photic layer than in the mesopelagic and the bathypelagic samples, while the phylogenetic diversity per iOTU and the MNTD was slightly higher in the bathypelagic layer (Fig. 2c).

**Table 1:** Summary of isolates, iOTUs, singletons and coverage per depth

	99% (non-rarefied)			99% (rarefied)		
	Photic	Mesopelagic	Bathypelagic	Photic	Mesopelagic	Bathypelagic
Number of isolates	817	362	382	346	362	368
Number of iOTUs	100	57	59	61	57	59
Number of Singletons	39	25	20	18	25	20
Good’s coverage	61%	56.1%	66.1%	70.5%	56.1%	66.1%

Results derived from isolates clustering at 99% sequence similarity to construct the non-rarefied and rarefied iOTU-abundance table (sampled down to the layer with the lowest number of isolates, i.e. mesopelagic with 362 isolates). Singletons: iOTUs appearing only once

### Shared diversity between photic, mesopelagic, and bathypelagic samples across oceans

**Fig. 3:**
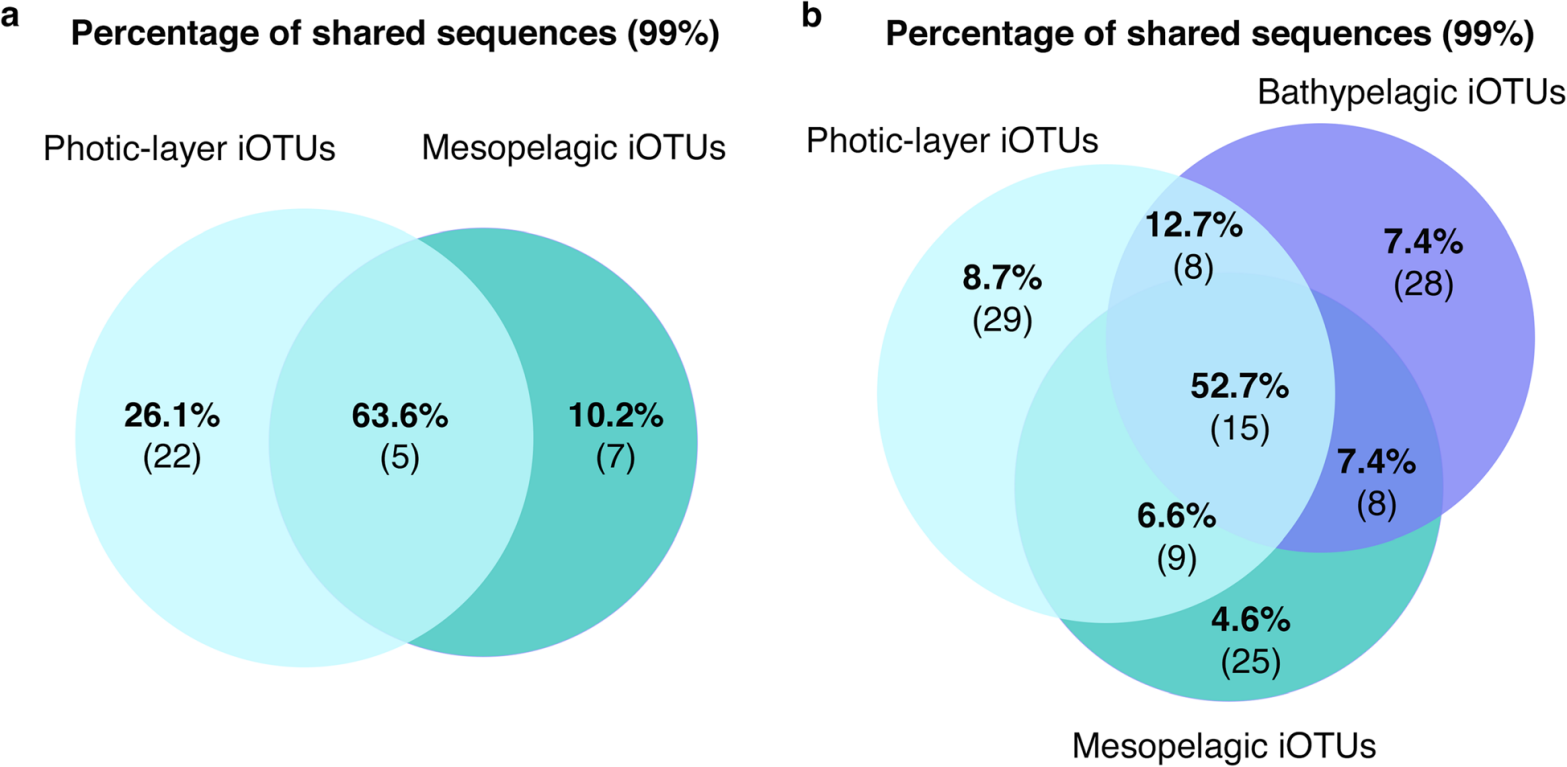
iOTUs retrieved from photic-layer and deep-sea waters. **a** Venn diagram representing the percentages of the sequences shared between photic and mesopelagic only from vertical profile samples of the station 39. Numbers inside brackets indicate the number of shared iOTUs corresponding to that percentage of sequences. **b** Venn diagram showing the percentages of the sequences shared between photic, mesopelagic and bathypelagic layers. Numbers inside brackets indicate the number of shared iOTUs corresponding to that percentage of sequences. Numbers displayed in all Venn diagrams are extracted from the rarefied iOTU-abundance tables

We explored the similarity between iOTUs from different layers (photic, mesopelagic and bathypelagic). First, we started with samples from Indian Ocean ST39 because it was the only station with a vertical profile covering samples of the photic (surface and deep chlorophyll maximum, DCM) and the aphotic layer (mesopelagic). A total of 34 iOTUs were obtained from the independent clustering at 99% sequence similarity of all isolates from ST39. This clustering revealed that only 5 iOTUs were shared between photic and mesopelagic, while the rest could only be recovered from one depth, being the photic layer with the highest number of different iOTUs (Fig. 3a), results that could be biased due to the higher presence of both surface and DCM isolates in the ST39 photic layer in comparison with the mesopelagic isolates. However, it was surprising that the shared iOTUs comprised the 63.6% of the total isolates (Fig. 3a). At this point, we also examined the connectivity between different layers and across distant oceans covering large spatial and latitudinal scales. The non-rarefied iOTU table, including all the photic (817), mesopelagic (362), and bathypelagic (382) isolates, as well as the rarefied iOTU table, sampled down to the layer with the lowest number of isolates (mesopelagic), were used for the analyses, and because minor differences were observed among them (Supplementary Tables S1 and S2 in Additional file 2), the results mentioned here refer only to those obtained after rarefaction. Fifteen out of 122 iOTUs (Fig. 3b) included isolates from all layers, accounting for 52.7% of the total isolates sequences (Fig. 3b), with an average number of 37.6 isolates per iOTU. Further, eight iOTUs (12.7% of the isolates) were common to photic and bathypelagic isolates, nine (6.6%) to photic and mesopelagic isolates, and eight (7.4%) to mesopelagic and bathypelagic isolates (Fig. 3b). Nevertheless, as observed in ST39, a substantial proportion of isolates were only retrieved from one of the layers: 29 iOTUs were only found in the photic samples, 25 in the mesopelagic, and 28 in the bathypelagic samples (Supplementary Table S3 in Additional file 2), with an average number of 3.2, 1.4, and 2.9 isolates per iOTU, respectively (Fig. 3b).

The taxonomic classification of all these iOTUs, using the lowest common ancestor (LCA) method, designated a total of 59 different genera and 10 iOTUs that could not be classified at the genus level. From these 59 genera, 13 were widely distributed along the different depths studied representing 75% of the total isolates. On the other hand, the photic ocean was again the layer with the highest number of retrieved genera that were not observed in the other two depths, even though they accounted for only 5.6% of the isolates (Supplementary Table S4 in Additional file 2).

If the comparative analysis is repeated with a more restrictive clustering, (instead of 99% at 100% similarity) we found that 37% of the isolates (578 out of 1561) were 100% identical at their partial 16S rRNA genes regardless the origin or layer. We found *Alteromonas*, *Cobetia*, *Erythrobacter*, *Leeuwenhoekiella, Halomonas*, *Idiomarina, Marinobacter,* and *Mesonia* between the shared genera, indicating taxa widely distributed along different depth layers. This shared percentage was even higher, up to 58.9%, when considering all mesopelagic and bathypelagic samples as aphotic and comparing them to all the photic isolates.

### Biogeography of the commonly isolated heterotrophic bacteria

**Fig. 4:**
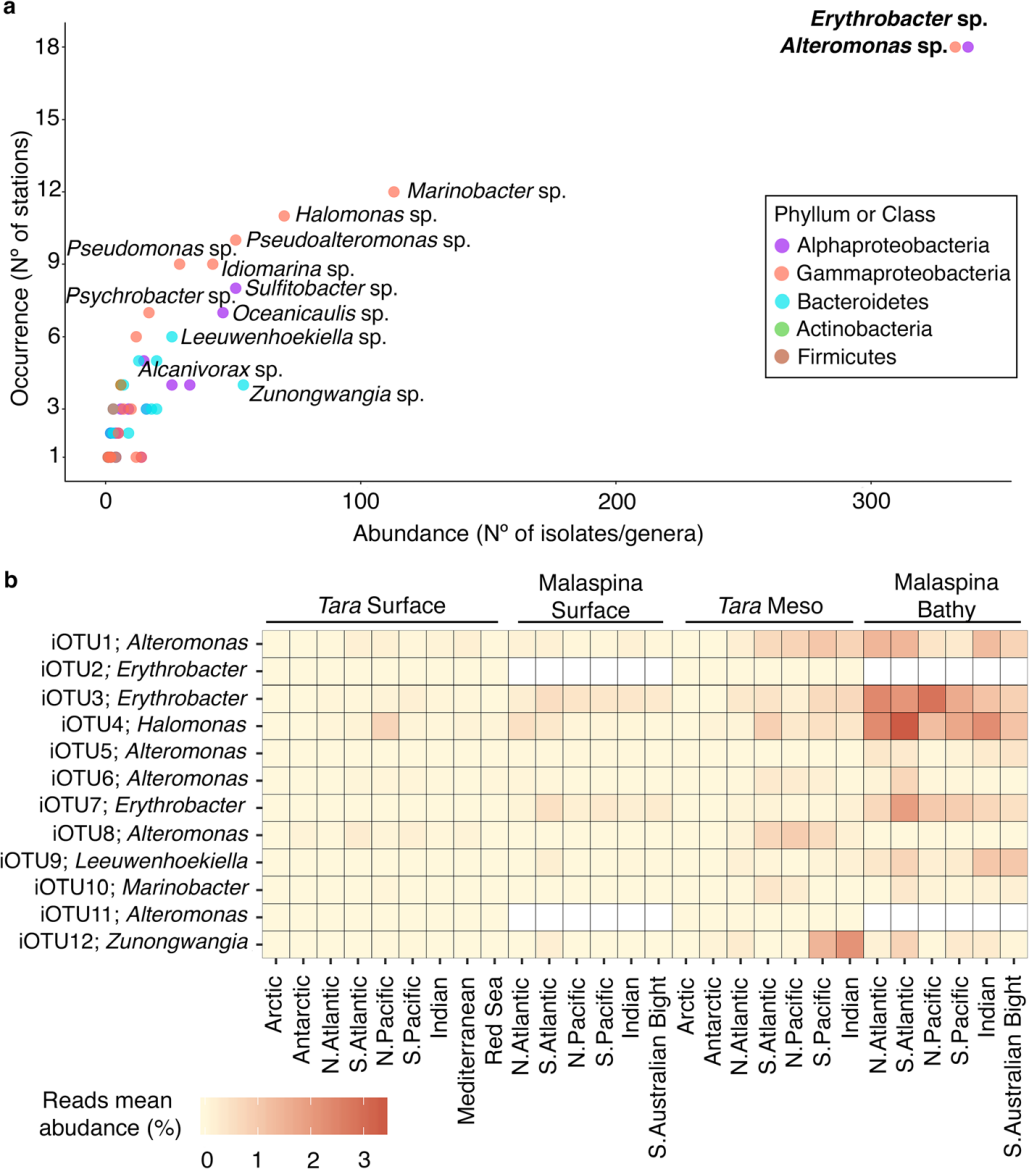
Abundance and biogeography of the isolates retrieved. **a** Abundance vs occurrence of the genera retrieved in the total culture collection. The most abundant and common genera are indicated in bold, and in regular type those with a more regional distribution. The color of the dots indicates the taxonomic (phylum or class) affiliation of the iOTUs. **b** Heatmap representing the mean abundance of reads (%) from zOTUs (zero-radius OTUs) of the top12 isolated OTUs (rows) along the different oceanographic regions studied in the *Tara* and Malaspina expeditions samples (columns). Subsampled zOTU-abundance tables from the different datasets have been used

The most abundant and common culturable genera, i.e. those that occurred in all or most (around 80%) of the 19 stations studied, and the ones only retrieved locally (around 25% of the samples) with a restricted distribution were identified. *Erythrobacter* and *Alteromonas* were the most abundant and recurrent genera retrieved, representing 41.3% of the isolates (338 and 333 isolates respectively), and appearing in 94% of the samples studied regardless their origin, season and year of sampling (Fig. 4a). Less abundant genera such as *Marinobacter* (113 isolates), *Halomonas* (70 isolates), *Pseudoalteromonas* (51 isolates), *Idiomarina* (42 isolates), *Pseudomonas* (29 isolates), *Sulfitobacter* (51 isolates), or *Oceanicaulis* (46 isolates) were present in more than 25% of the samples (Fig. 4a) and covered almost all the oceanographic regions (Supplementary Table S5 in Additional file 2). These could be considered, thus, regionally distributed. Some genera such as *Psychrobacter*, *Leeuwenhoekiella* or *Alcanivorax* had lower numbers of isolates, but were recovered in more than 25% of the samples (Fig. 4a). Other genera, in turn, such as *Zunongwangia,* were retrieved in less than 25% of the samples but presented 54 isolates (3.5% of the strains). All the mentioned genera were found in the photic, mesopelagic, and bathypelagic layers, except *Oceanicaulis* which could not be isolated in this study from the bathypelagic samples (Supplementary Table S5 in Additional file 2). Finally, the remaining genera represented 20% of the cultures. Then, these results revealed which genera are commonly isolated from distant stations with contrasted environmental conditions, depths and seasons covering 6 years of temporal range.

In a parallel study, we compared the isolates from each station with 16S rRNA sequences obtained through Illumina HTS of environmental DNA (16S iTAGs, hereafter) from two marine circumnavigations (*Tara* Oceans [28] and Malaspina Expedition [29]), to investigate whether our isolates have identical matches with environmental 16S iTAGs (Sanz-Sáez et al. in preparation). Despite the global comparison is out of the scope of this study, here we present the biogeographic distribution of the abundant top12 iOTUs, those with more than 20 isolates (Supplementary Table S6 in Additional file 2). To do so, we show the relative abundances of the denoised zOTUs (zero-radius OTUs, i.e. OTUs defined at 100% sequence similarity), obtained from the 16S iTAGs that matched at 100% similarity with these top12 iOTUs. We were aware that different iOTUs could match with the same zOTUs, and therefore the biogeography results presented here for the different zOTU represent the sum of the abundances of the top12 abundant iOTUs with other less abundant/rare iOTUs (Supplementary Table S7 in Additional file 2). The top12 iOTUs matching at 100% with zOTUs represented the 48.3% of the total isolates (754 out of 1561), and only one of the zOTUs matched with two iOTUs, a top12 iOTU and a less abundant/rare iOTUs, the latter representing 6 out 754 (0.8%) of the isolates included in the top12 iOTUs. Besides, both iOTUs matching to the same zOTUs affiliated with the same genus and, thus, the abundance presented would correspond to different species or ecotypes within a genus. Thereby, in the photic layer, the abundant top12 iOTUs, or in this case, their respective zOTUs matches, represented an average abundance of 16S iTAGs (at 100% similarity) lower than 1%, regardless of their geographic region (Fig. 4b). This percentage increased around 1–2% of the reads in the mesopelagic layer in specific regions such as the Indian and South Pacific Ocean. However, our isolates exhibited higher abundances in the bathypelagic layer, in almost all oceanographic regions, especially for iOTU1, iOTU3 and iOTU4 affiliating with *Alteromonas*, *Erythrobacter* and *Halomonas* spp. (Fig. 4b). Overall, these iOTUs accounted a total average proportion of reads of 0.3 and 1.1% in the photic layer in two independent datasets (*Tara* and Malaspina respectively), 2.7% in the mesopelagic layer, and up to 7.8% in the bathypelagic, indicating that the commonly found isolates are more abundant in the deeper layers of the ocean.

### Novelty of the isolates of the MARINHET collection

**Fig. 5:**
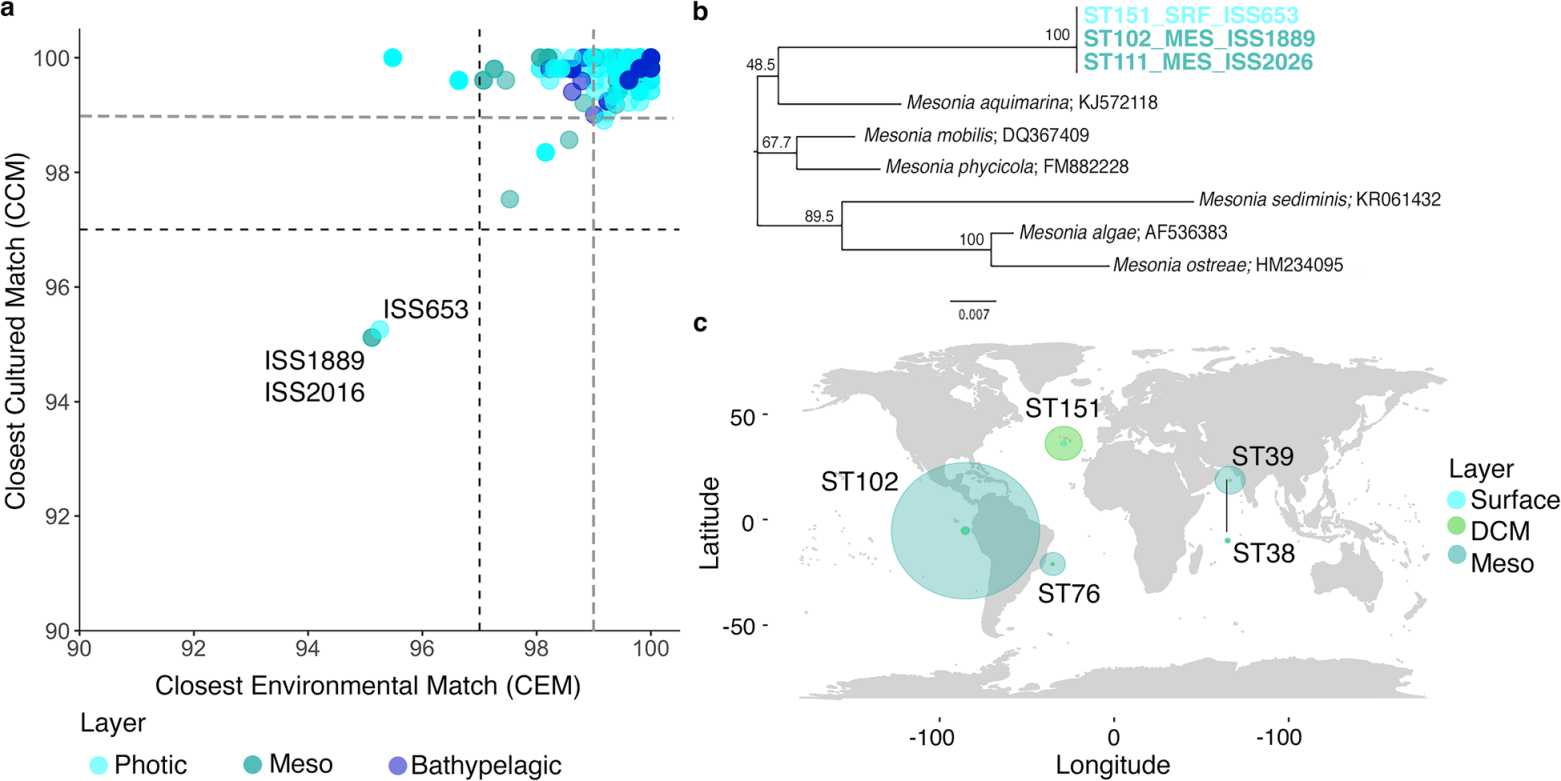
Potential novel isolates. **a** Percentages of similarity between the Closest Cultured Match (CCM) and the Closest Environmental Match of all the 16S rRNA gene sequences. Horizontal and vertical lines represent the typical cut-off value of 97% (black dashed lines) and 99% (grey dashed lines) commonly used for “species” delineation. **b** Neighbour Joining tree of the putative *Mesonia* isolates. The numbers in the nodes represent bootstrap percentages > 45%, calculated from 1000 replicates. Putative new isolates are written in bold letters and color indicates origin of the isolates. **c** Read recruitment of ISS653 and ISS1889 in 5 *Tara* Oceans stations. They include the stations where the isolates were retrieved (ST151 and ST102) and some distant stations for the sake of comparison (ST39, ST38, ST76). ST38 is located near ST39 (Latitude 19° 2.24′ N, Longitude 64° 29.24′ E), but its location in the plot was slightly modified for its correct visualization. Size of the circles are the sum of the abundance of reads from both genomes recruited in each station and layer (x10k). SRF, surface isolates; DCM, deep chlorophyll maximum; Meso, mesopelagic isolates

The percentages of similarity between the strains and their Closest Cultured Match (CCM) and Closest Environmental Match (CEM) were extracted and compared with the 97 and 99% identity thresholds to explore the possible novelty of our culture collection. The results showed that most of the isolates were similar to previously published cultured bacterial species, but also to environmental sequences obtained using molecular techniques (Fig. 5a). Therefore, most of the isolates were previously known microorganisms. However, we detected three 100% identical strains in their partial 16S rRNA gene that had a percentage of identity, both for CCM and CEM, below the threshold, at around 94%. One of the strains was isolated from surface samples of the North Atlantic Ocean (ISS653), whereas the other two were isolated from two mesopelagic samples of the Pacific Ocean (ISS1889, ISS2026). Further analyses with the complete 16S rRNA gene of ISS653 indicated that they could be candidates for a new species or even a new genus according to the thresholds proposed by Yarza et al. [30]. The three databases consulted (National Center for Biotechnology Information (NCBI), Ribosomal Data Base Project (RDP) and SILVA showed different BLASTn results (Supplementary Table S8 in Additional file 2). Nevertheless, the Living Tree Project (LTP) database, which contains the accepted type species of each genus, displayed a 93.5% similarity with *Mesonia mobilis*. The phylogenetic tree constructed (Fig. 5b) also supported its novelty as our isolates had less than 93.8% of similarity with the cultivated reference genomes of the *Mesonia* genus.

Genomes of the strains ISS653 and ISS1889 were fully sequenced and characterized to formally describe a novel species, *Mesonia oceanica* [80]. As a detailed description of the novel species is already given in Lucena et al. [80], here we only focused in some interesting phenotypic differences among these two strains and their distribution pattern in marine metagenomes from five stations, including ST102 and ST151, that were the ones in which ISS1889 and ISS653 were respectively isolated. Only a few phenotypic differences could be found among both strains (Table 2). The most important phenotypic trait was the difference in their maximum growth temperatures, being 37 °C for ISS653, isolated from surface waters in ST151, and 30 °C for ISS1889, isolated from the mesopelagic layer in ST102. Genomic comparisons of both strains revealed an average nucleotide identity (ANI) of 99.9%, which indicated that the two strains were almost identical genetic clones. The G + C content and the number of RNAs present were equal in both strains. They slightly differ in the size and the number of protein codifying sequences (Table 2). However, we identified a pool of unique genes for each strain, 33 were only found in ISS653, whereas 6 were unique in ISS1889 (Supplementary Table S9 in Additional file 2). Among them, we found interesting some proteins that may confer specific advantages and/or adaptation (Table 2). For example, ISS653 contains some chaperones, GroEL, GroES and ClpB, that may be related with its wider range of growth temperatures. In addition, we detected some resistant mechanisms to toxic heavy metals. Resistance genes to cadmium-zinc-cobalt were detected in ISS653, while mercury resistance genes were observed in ISS1889. Nevertheless, we found that both isolates presented the same distribution patterns (Fig. 5c and Supplementary Fig. S2 in Additional file 1). Thus, these two new strains displayed higher abundances in the mesopelagic waters regardless of the station analysed, but especially in ST102 where ISS1889 was retrieved (Fig. 5c). Strain ISS653 was isolated from surface waters of the ST151 but it was in deeper layers where its abundance was also higher (Supplementary Fig. S3 in Additional file 1).

**Table 2:** Origin of the novel *Mesonia* strains ISS653 and ISS1889 and phenotypic and genetic main differences.

	ISS653	ISS1889
Station	ST 151	ST 102
Ocean	North Atlantic Ocean	South Pacific Ocean
Depth (m)	5	475.6
In situ Temperature (°C)	17.3	9.2
Physiology differences:		
Maximum temperature for growth (°C)	37	30
Tween-80 hydrolysis	weak	–
Phenylacetate assimilation (API20NE)	+	–
Acid from (API50CH/E):		
arbutin	–	weak
2-ketoglutarate	–	weak
Cellular fatty acids:		
iso-C_15:0_ 2OH	11.2	14.5
iso-C_17:0_ 3OH	9.2	14.6
iso-C_17:1_ ω***9c***	5.2	10.5
C_15:1_ ω***5c***	3.6	Traces (< 1%)
C_18:1_ ω***c***	3.9	–
Genomic differences:		
Genome size (bp)	4,275,762	4,283,636
G + C content (mol%)	34.9	34.9
RNAs	45	45
Protein codifying sequences:	4030	4015
Chaperones	GroEL, GroES, ClpB	–
Cobalt-zinc-cadmium resistance	CusA, CzcA, CzcD	–
Mercury resistance	–	MerA, MerT

Differences extracted from a total of 158 tests (Supplementary Methods in Additional file 1). Within protein codifying sequences we included a list of some interesting proteins that were unique for one of the strains

## Discussion

We have elaborated an extensive marine heterotrophic bacterial culture collection with 1561 isolates covering different oceanographic regions, depths, seasons and years. We used an standard marine medium to reach the heterotrophic fraction of the community that could be comparable between layers (photic and aphotic) and across oceans, rather than using other specific media for increasing the novelty on isolates in the deep ocean. Even though we could not fully address distribution patterns along complete latitudinal gradients for all depth layers studied or some seasonal/temporal changes, we could explore the phylogenetic diversity of the MARINHET culture collection and analyse the potential differences between depths. The alpha-diversity metrics were slightly higher in the photic layer, but not significant differences between layers were found. On the other hand, rank abundance curves from different depths showed that the fraction of the heterotrophic isolates retrieved were composed by a few abundant iOTUs with a large number of representatives and many rare low abundant iOTUs, which, in this case, is consistent with many other previous findings based on prokaryotic amplicon 16S iTAGs from environmental samples [1, 31]. For instance, the 7 most abundant iOTUs (99%) accounted for 41% of the total isolates and similar proportions were found in each layer. Hence, 30% of the bathypelagic isolates, 47% of the mesopelagic isolates, and 43% of the photic isolates affiliated with these seven most abundant iOTUs.

The comparison between those isolates coming from different depths allowed us to detect certain level of vertical connectivity among the heterotrophic culturable community. The significant overlap found between photic, mesopelagic and bathypelagic strains suggest that these heterotrophic bacteria are well adapted to different temperatures, light and pressure. Moreover, they probably have versatile metabolisms to respond to different environments and nutrient availability. These characteristics may make these bacteria more prone to successfully face such long vertical and horizontal dispersion [32]. In addition, genomic comparison between cultured isolates and uncultured genomes retrieved by single amplified genomes (SAGs) from marine environments revealed that the genomes of the cultures had larger sizes, suggesting a predominant copiotrophic lifestyle [33]. One possible explanation supporting the high proportion of identical 16S rRNA gene sequences between isolates of photic and aphotic layers, up to 58.9%, would be that these bacteria have the capacity to attach and grow on particles in the photic layers and after sinking to the deep ocean, they still retain the capability for further growth. Certainly, a recent study claimed that the particle colonization process that takes place in the photic layers determines the composition of deeper layers and especially bathypelagic communities, and thus, photic and deep-ocean prokaryotic communities are strongly connected via sinking particles [34]. Moreover, the attachment to particles and its presence in the deep ocean has been described at least for *Alteromonas* [34–37], *Erythrobacter* and *Halomonas* [34].

Those mentioned genera are also the most abundant and commonly isolated in all depths of our dataset together with *Marinobacter*. These genera have been detected in other culture-dependent and culture-independent studies from a wide variety of marine environments, including coastal, shelf, and open ocean waters [4, 37–42] corroborating their ubiquity. *Alteromonas* and *Erythrobacter* presented the highest number of isolates. *Alteromonas* is among the most common culturable heterotrophic bacteria living in open marine waters all around the world, as it has been isolated from a wide variety of marine environments [8, 43–46]. In addition, this genus is thought to be one of the most significant contributors of the dissolved organic carbon (DOC) consumption and nutrient mineralization in the upper ocean [47]. *Erythrobacter* strains are aerobic chemoorganotrophs, and some species contain *bacteriochlorophyll a*, responsible for the aerobic anoxygenic phototrophic (AAP) metabolism [40].

Despite these findings, one of the remaining questions, mainly in relation to the commonly isolated bacteria, is to what extent these strains match with environmental 16S rRNA genes from HTS sequencing of the whole bacterial community. The comparison of the top abundant iOTUs sequences with 16S iTAGs confirmed that these common iOTUs matched at 100% identity with environmental sequences at different extent, being rare at the surface but with increasing representation in the deep ocean, especially in the bathypelagic (Sanz-Sáez et al., in preparation).

On the other hand, even though the isolation of novel strains was a secondary objective, thanks to the large isolation effort done, we managed to isolate three strains, 100% similar among them in their partial 16S rRNA gene, that presented less than 95% of similarity in their 16S sequence to any previously described bacterial species. There are several well-accepted criteria for the classification of bacteria into species and one of them is based in the 16S rRNA gene sequence identity threshold at around 98.7–99% [30, 48, 49]. Two of these strains, ISS653 and ISS1889, are being now fully characterized and their genome has been sequenced by the Spanish Culture Collection of Type Strains (CECT) to formally describe a novel species, *Mesonia oceanica* [80]. Members of this taxon are mainly retrieved from a variety of marine environments, sometimes associated with eukaryotic organisms, such as algae [50]. Interestingly, isolate ISS653 was obtained from surface North Atlantic waters whereas ISS1889 was retrieved from mesopelagic waters of the Pacific Ocean, and the biogeography analysis in some vertical profiles suggest that this putative novel species is not locally restricted and it has preference for deeper layers. Curiously, even though we could not detect any significant difference in the distribution of these two strains and genetically they seem almost clonal, we cannot discard that their minimal genetic differences and phenotypic plasticity may provide adaptation advantages (of growth rate and tolerance to metals) under particular environmental conditions not observed in our marine samples.

Finally, as we stated at the beginning of this study, cultures are important because they allow to retrieve novel bacterial taxa and complete genomes, but most importantly, they enable to test hypotheses that emerge from genomic data. However, the isolation of a great battery of strains from different oceanographic regions and depths by traditional culture techniques, as we presented, does not guarantee the retrieval of many new bacterial species or taxa detected by HTS techniques, which greatly outnumber those accessible by cultivation. One of the challenges that marine microbial ecologists still face is the innovation in the isolation methods for the retrieval of axenic cultures of those uncultured taxa. In the last years, new isolation approaches had been developed to improve the recovery of bacteria under laboratory conditions like microfluidics [51, 52], cultivation chips [53–55], microcolony cultivation techniques [56], manipulation of single cells [57, 58], and high-throughput cultivation techniques named “culturomics” [59, 60]. Nevertheless, all these strategies are usually expensive and include inherent trial-and-error approaches. In this manner, metagenomic, metatranscriptomic and metaproteomic data, which had increased our knowledge of the microorganisms present in marine ecosystems and allowed to predict their metabolic capabilities, would provide essential information to design different isolation strategies and allow the retrieval of environmental bacteria. Accordingly, understanding the microbial complexity of the marine ecosystems would be possible if combined culture-dependent and culture-independent studies start to be the rule among marine microbial ecologists.

## Conclusions

In summary, culturing remains an important tool in microbial ecology, helping to map the diversity of marine communities. We are aware that our study is restricted to those heterotrophic marine bacteria that can grow in standard culture conditions, and that we are missing many other fundamental microbial populations that do not grow easily in standard marine media. Nevertheless, given the important isolation effort done and the number of oceanic regions and depths covered in different years, we were able to enhance our knowledge of the taxonomy, phylogenetic diversity and distribution of the targeted bacteria. Equally to those HTS studies of ribosomal genes targeting the whole marine prokaryotic community, the culturable marine heterotrophic bacteria isolated presented few abundant taxa and a tail of rare and low abundant iOTUs. We detected that half of the total isolates were shared in the three different depth realms, reinforcing the already introduced idea of vertical connectivity between the photic and the deep ocean probably through sinking particles. In addition, we identified *Alteromonas* and *Erythrobacter* genera to be the most abundant and commonly isolated heterotrophic bacteria from more than 80% of the studied samples and from all layers*.* Finally, we found three strains belonging to a new species of the genus *Mesonia*. Overall, this study highlights the relevance of complementary studies with focus on marine isolated bacteria to provide a more comprehensive view of marine microbial diversity. Furthermore, our MARINHET culture collection represents a valuable resource for future genome sequencing projects and potential physiological experiments involving marine isolates.

## Methods

### Study areas and sampling

A total of eight photic-layer, four mesopelagic, and seven bathypelagic samples were taken during different oceanographic cruises in several sampling stations distributed along a wide range of latitudes (Fig. 1). Photic-layer samples (Table 3) were collected in the Atlantic and Indian Oceans during the *Tara* Oceans expedition in 2009–2013 [28], and from the Arctic Ocean during the ATP cruise in 2009 [61]. Additionally, surface seawater samples from the Blanes Bay Microbial Observatory (BBMO, http://www.icm.csic.es/bio/projects/icmicrobis/bbmo) in the NW Mediterranean Sea were collected in May 2015. Mesopelagic samples (Table 3) were taken from the Indian and the Pacific Oceans also during the *Tara* Oceans expedition in 2009–2013 [28]. All the mesopelagic samples were collected in regions with oxygen minimum zone (OMZ) areas, ST39 from the Arabian Sea, ST102 and ST111 from the Eastern Tropical South Pacific, and ST138 from the Eastern Tropical North Pacific. Bathypelagic samples (Table 3) from the Atlantic Ocean at ~ 4000 m depth were taken from six different stations during the Malaspina 2010 Circumnavigation Expedition [29]. One of the stations sampled was located in the North Atlantic, whereas the other five stations were located in the South Atlantic. ST43 could specially differ from the rest South Atlantic samples because it was particularly placed in the Agulhas Ring, where deep waters from the South Atlantic converge and mix with Indian Ocean deep-water masses [62]. In addition, one bathypelagic sample was collected at 2000 m depth in the NW Mediterranean during the MIFASOL cruise in September 2014.

**Table 3:** Characteristics of the different samples used for isolation of marine heterotrophic bacteria

Cruise	Station	Sampling date	Oceanic location	Latitude	Longitude	Depth (m)	In situ temperature (°C)	N° of sequenced isolates	N° of non-redundant isolates^**a**^
*Tara* Oceans	ST 39	March 2010	Indian Ocean	18° 35.2′ N	66° 28.22′ E	5.5	26.2	104	25
	ST 39	March 2010	Indian Ocean	18° 35.2′ N	66° 28.22′ E	25	26.8	243	53
	ST 39	March 2010	Indian Ocean	18° 43.12′ N	66° 21.3′ E	268.2	15.6	88	18
	ST 67	September 2010	South Atlantic	32° 17.31′ S	17° 12.22′ E	5	12.8	115	49
	ST 72	October 2010	South Atlantic	8° 46.44′ S	17° 54.36′ W	5	25	71	33
	ST 76	October 2010	South Atlantic	20° 56.7′ S	35° 10.49′ W	5	23.3	89	27
	ST 151	March 2012	North Atlantic	36° 10.17′ N	29° 1.23′ W	5	17.3	76	33
	ST 102	April 2011	Pacific Ocean	5° 16.12′ S	85° 13.12′ O	475.6	9.2	97	15
	ST 111	June 2011	Pacific Ocean	16° 57.36′ S	100° 39.36′ O	347.1	10.9	98	35
	ST 138	December 2011	Pacific Ocean	6° 22.12′ N	103° 4.12′ O	444.9	8.2	79	34
ATP	AR_1	June 2009	Arctic Ocean	78° 20.00′ N	15° 00.00′ E	2	6.2	13	9
	AR_2	June 2009	Arctic Ocean	76° 28.65′ N	28° 00.62′ E	25	−1.2	20	9
Malaspina	ST 10	December 2010	North Atlantic	21° 33.36′ N	23°26′ W	4002	2	20	9
	ST 17	February 2011	South Atlantic	3° 1.48′ S	27° 19.48′ W	4002	1.7	93	24
	ST 23	August 2011	South Atlantic	15° 49.48′ S	33° 24.36′ W	4003	1.5	94	39
	ST 32	January 2011	South Atlantic	26° 56.8′ S	21° 24′ W	3200	2.5	39	16
	ST 33	January 2011	South Atlantic	27° 33.2′ S	18° 5.4′ W	3904	1.7	5	5
	ST 43	April 2011	South Atlantic	32° 48.8′ S	12° 46.2′ E	4000	1.2	4	4
MIFASOL	ST 8	September 2014	NW Mediterranean	40° 38.41′ N	2° 50′ E	2000	13.2	127	36
BBMO	IBSURF	May 2015	NW Mediterranean	41° 40′ N	2° 48′ E	5	17.7	86	43

^a^Non-redundant isolates stand for the number of different isolates remaining after removing those that were 100% identical in their partial 16S rRNA gene

In each of these stations, seawater was collected using Niskin bottles attached to a rosette sampling system, except at BBMO, where samples were collected with a bucket. Seawater was sequentially filtered through 200 μm and 20 μm meshes to remove large plankton cells and to keep the free-living bacterial community together with the one attached to particles (< 20 μm). Duplicate 2 ml seawater of each station were kept in Eppendorf tubes with dimethyl sulfoxide (DMSO) 7% final concentration and stored at − 80 °C until further processing in the laboratory.

Geographical coordinates of stations, sampling date, sampled depth, in situ temperature and total number of sequenced isolates are listed in Table 3.

### Culturing and isolation

Isolates were obtained by plating 100 μl of undiluted and 10x diluted seawater from the photic, mesopelagic and bathypelagic samples, in triplicates, onto Zobell agar plates (i.e. 5 g peptone, 1 g yeast extract and 15 g agar in 750 ml of 30 kDa filtered seawater and 250 ml of Milli-Q water) or Marine Agar 2216 (Difco™) plates, which is based also on the Zobell medium formulation [63]. Our medium culturing strategy was only focused to retrieve heterotrophic marine bacteria that could grow easily under laboratory conditions (nutrient rich medium, standard oxygen concentrations and atmospheric pressure) using two similar culturing media. The only difference between Zobell agar and Marine Agar 2216 plates is the use of natural seawater (Zobell agar), or the addition of the minerals and salts contained in natural seawater to distilled water (Marine Agar 2216). Indeed, we did not observe significant differences (Fisher test analyses, data not shown) in the bacterial isolation between both media.

Photic-layer and mesopelagic samples were incubated at room temperature (RT, ~ 20 °C) while bathypelagic samples were incubated at their in situ temperature, which ranged from ~ 4 °C (in the Atlantic Ocean at 4000 m depth) to 12 °C (NW Mediterranean at 2000 m depth) (Table 3 and Supplementary Table S10 in Additional file 2), but also at RT in order to assure bacterial recovery from all stations. In all cases, triplicates of each temperature condition and dilution were incubated in the dark until no more colonies appeared (10–30 days).

A total of 1561 bacterial isolates were randomly selected for DNA amplification and partial sequencing of their 16S rRNA gene (Table 3 and details below). Similar number of isolates were sequenced from photic layers (817; average: 102 isolates per station) and from deep oceans (744; average: 67 isolates per station) with 362 isolates from the mesopelagic and 382 from the bathypelagic. In most of the bathypelagic samples we collected all colonies appearing in the plates, which ranged from 6 to 129 including all replicates. Colonies were streaked on agar plates in duplicate to ensure their purity and avoid contamination. The isolates were stored in the broth medium used with glycerol 25% in cryovials at − 80 °C.

### PCR amplification and sequencing of the 16S rRNA gene

Available DNA used for template in Polymerase Chain Reaction (PCR) was extracted from 200 μL of isolates liquid cultures placed in 96 well plates, diluted 1:4 and heated (95 °C, 15 min) to cause cell lysis. The partial 16S rRNA gene sequences were PCR amplified using bacterial primers 358F (5′-CCT ACG GGA GGC AGC AG-3′) [64] and 907Rmod (5′-CCG TCA ATT CMT TTG AGT TT-3′) [65]. The complete 16S rRNA gene was amplified for *Mesonia* strain ISS653 after DNA extraction using the DNeasy Blood & Tissue kit (Qiagen), following the manufacturer’s recommendations, and using the modified primers from Page et al. [66] 27F (5′- AGR GTT TGA TCM TGG CTC AG − 3′) and 1492R (5′- TAC GGY TAC CTT GTT AYG ACT T − 3′). Detailed PCR conditions are described in Supplementary Methods (Additional file 1). Purification and OneShot Sanger sequencing of 16S rRNA gene products was performed by Genoscreen (Lille, France) with primer 358F for partial sequences, and with both 27F and 1492R for complete sequences. ChromasPro 2.1.8 software (Technelysium) was used for manual cleaning and quality control of the sequences.

### Data processing and taxonomic classification

The 16S rRNA sequences of our cultured strains were clustered at 99% sequence similarity [48] in order to define different operational taxonomic units (iOTUs or isolated OTUs) and construct iOTU-abundance tables for the different stations and layers studied (Supplementary Table S6 in Additional file 2) using UCLUST algorithm from the USEARCH software [67]. The different iOTUs were taxonomically classified using the lowest common ancestor (LCA) method in SINA classifier [68], using both SILVA (release 132 in 2017) and RDP (Ribosomal Database Project, release 11) databases. Parallelly, isolates sequences were submitted to BLASTn [69] with two subsets of the RDP database, one including only the uncultured bacteria (Closest Environmental Match, CEM), and another including only the cultured bacteria (Closest Cultured Match, CCM) in order to extract the percentages of similarity with both datasets (Supplementary Tables S11 and S12 in Additional file 2), and to assess whether our isolates were similar to effectively published cultured organisms.

Additionally, a more restrictive clustering at 100% sequence similarity (USEARCH software) was also used to define iOTUs and to detect how many bathypelagic, mesopelagic and photic-layer bacterial isolates were identical, and thus, to identify bacterial taxa or strains that could distribute along different water depths. Such comparisons were done with: (i) photic and mesopelagic isolates sequences retrieved from the ST39 vertical profile and (ii) the whole isolates dataset.

### Phylogenetic analyses

The phylogeny was inferred for the representative isolates of each iOTU defined at 99 and 100% sequence similarity. The closest sequence to each isolated iOTU in SILVA v.132 database was found and collected using BLASTn [69]. Alignment of the isolates and reference sequences was performed with MUSCLE from the Geneious software v.11.0.5 [70]. The alignment was trimmed to the common 16S rRNA gene fragment covered by both sets of sequences. Phylogeny was constructed using maximum-likelihood inference with RAXML-NG 0.9.0 [71] and the GTR evolutionary model with optimization in the among-site rate heterogeneity model and the proportion of invariant sites (GTR + G + I), and 100 bootstrap replicates.

Eventually, some isolates among our culture collection presented partial 16S rRNA sequences with a percentage of similarity below the 97% with public databases. In this case, the complete 16S rRNA gene was sequenced for ISS653, with which two more strains (ISS1889 and ISS2026) clustered at 100% similarity, and a phylogenetic tree was constructed to support its putative novelty. The tree included their complete and partial sequences of the 16S rRNA gene, their best hits from uncultured and cultured microorganisms, extracted from local alignments against RDP 11, SILVA LTP (Living Tree project), and National Center for Biotechnology Information (NCBI) databases, and the reference 16S rRNA genes from their related genera. Details on the phylogenetic tree constructions are explained in Supplementary Methods (Additional file 1).

### Comparisons between layers and statistical analyses

All data treatment and statistical analyses were conducted in the R statistical software version 3.4.3 [72] and packages *stats, vegan* version 2.5–3 [73]*, ape* version 5.1 [74]*, picante* version 1.6–2 [75] and *EcolUtils* [76]. In general, analyses were performed using the non-rarefied iOTU-abundance tables, but for specific analyses, such as the detection of iOTUs present along different depths, the iOTU-abundance table constructed with the sequences clustering at 99% was sampled down to the lowest sampling effort (362 isolates in the mesopelagic). In this manner, the rarefied or subsampled iOTU table was obtained using the function *rrarefy.perm* with 1000 permutations from the R package *EcolUtils* [76].

Rarefaction curves were performed with the package *vegan* to estimate the sampling effort in each studied layer. We also calculated bacterial richness/diversity metrics from each depth using two approaches: an OTU-based approach (i.e. considering the iOTUs as unrelated biological entities), and a phylogenetic approach (i.e. considering the evolutionary relationships among iOTUs with the complete computed phylogeny). The number of iOTUs, the Chao extrapolative richness estimator [77] and the Shannon entropy index [78] were computed as OTU-based metric using the non-rarefied iOTU abundance table, while the Faith’s phylogenetic diversity (PD) [26], the PD divided by the number of iOTUs (PD/iOTUs), and the mean nearest taxon distance (MNTD) [27] were used as phylogenetic measures for diversity. Differences between photic, mesopelagic and bathypelagic for richness/diversity measures were tested using an ANOVA test followed by the Tukey’s post hoc test, as data normality was assured. To assess significance, the statistical analyses were set to a conservative alpha value of 0.01.

The Good’s coverage (C) for each of the depths was also calculated by the equation $C=\left[1-\left(\frac{n_1}{N}\right)\right]\ast 100\%$, where *N* is the number of iOTUs being examined and *n*_*1*_ represents the number of iOTUs occurring only once or singletons [79].

### Comparison to environmental 16S rRNA Illumina sequences

Isolates were compared to denoised zOTUs (zero-radius OTUs, i.e. OTUs defined at 100% sequence similarity) [67] from high-throughput sequencing (HTS) of the 16S rRNA sequences (16S iTAGs) obtained from *Tara* Oceans and Malaspina Expedition datasets which covered surface, mesopelagic and bathypelagic layers. Further description of those datasets, sample collection, DNA extraction, sequence processing and data treatment are described in Supplementary Methods (Additional file 1). All isolates sequences were compared to zOTUs sequences at 100% similarity respectively, by running global alignment using the -*usearch_global* option from the USEARCH v10.0.240 [67]. The results were filtered by coverage of the alignment at 100% and in those cases where isolates had more than one hit, only the ones with the higher percentage of identity were kept. Primers used to obtain the 16S rRNA genes of the isolates were different from the ones used to obtain the 16S rRNA iTAGs, but both amplified the V4 and V5 hypervariable region of the gene, so comparisons could be done by this method. For each dataset compared we calculated the mean percentage of reads or iTAGs, and zOTUs of the bacterial community that matched at 100% similarity with the 16S rRNA sequences of the strains isolated by traditional culture techniques. These percentages were calculated from the rarefied zOTU-abundance tables.

### Genomes of ISS653 and ISS1899 and fragment recruitment analysis in marine metagenomes

Genomes of ISS653 and ISS1889 were sequenced and analysed by the Spanish Culture Collection of Type Strains (CECT). The accession number of ISS653 16S rRNA gene sequence and draft genome are MH732189 and CABVMM01, respectively, while the accession number of ISS1889 16S rRNA gene is MN836382. Detailed description of genome sequencing and analyses can be found in Lucena et al. [80]. Metagenomic reads from some selected *Tara* Oceans stations (ST38: SUR (surface), DCM (deep chlorophyll maximum), MES (mesopelagic); ST39: DCM, MES; ST76: SUR, DCM, MES; ST102: SUR, DCM, MES; and ST151: SUR, DCM) were recruited competitively against the pool of the assembled contigs of the two isolates genomes. All metagenomes were subsampled to the shallower sequencing depth (129,995,612 fragments; mesopelagic from ST38) with *bbtools reformat*.sh (v38.08; https://sourceforge.net/projects/bbmap/). BLASTn v2.7.1+ [69] was used to map the reads with the following alignment parameters*: -perc_identity* 70, *−evalue* 0.0001. Only those reads with more than 90% coverage and mapping at identities equal to or higher than 95% were considered to be true positives. In order to remove possible false hits mapping to the conserved regions of rRNA genes, reads aligning to the regions annotated as ribosomal genes were not considered for the analysis. Reads mapping with the same probability to any of the genomes were assigned at random.

### Nucleotide sequences accession number

The 16S rRNA gene sequences of the bacterial isolates retrieved in this study were deposited in GenBank. Sequences from all isolates, except those coming from the mesopelagic regions and those from the surface Indian Ocean, were deposited under accession numbers MH731309 - MH732621. Notice that among these accession numbers other isolates are included, originated from the same locations but isolated with another culture medium and not included in this study. Isolates retrieved from the mesopelagic and those from the surface Indian Ocean are deposited under accession numbers MK658870-MK659428.

## Supplementary information

**Additional file 1.** Includes Supplementary Methods, Supplementary Figures and Supplementary Tables headings.

**Additional file 2.** Includes all Supplementary Tables.

## Data Availability

The isolates sequence dataset generated and analysed during the current study are deposited in GenBank under the accession numbers MH731309 - MH732621 and MK658870-MK659428. The *Tara* and Malaspina 16S iTAGs datasets analysed during the current study are available from the corresponding author on reasonable request.

## Abbreviations

ANI: Average Nucleotide Identity
BBMO: Blanes Bay Microbial Observatory
CECT: Colección Española Cultivos Tipo
CEM: Closest Environmental Match
CCM: Closest Cultured Match
DCM: Deep Chlorophyll Maximum
DMSO: Dimethyl sulfoxide
DOC: Dissolved Organic Carbon
HTS: High-throughput sequencing
iOTUs: Isolates Operational Taxonomic Units
LCA: Lowest Common Ancestor
LTP: Living Tree project
MARINHET: MARIN HETerotrophic bacterial culture collection
MNTD: Mean Nearest Taxon Distance
NCBI: National Center for Biotechnology Information
OMZ: Oxygen Minimum Zone
PCR: Polymerase Chain Reaction
PD: Faith’s Phylogenetic Diversity
RDP: Ribosomal Database Project
RT: Room Temperature
SAGs: Single Amplified Genomes
zOTUs: Zero-radius Operational Taxonomic Units
16S iTAGs: 16S illumina TAGs
