# Supplementary material for: Diversity and distribution of marine heterotrophic bacteria from a large culture collection
Source: BMC Microbiol. 2020 Jul 13;20:207. doi: 10.1186/s12866-020-01884-7 (PMC7359222; doi:10.1186/s12866-020-01884-7)
Supplement: Supplementary file 1 — Additional file 1. Includes Supplementary Methods, Supplementary Figures and Supplementary Tables headings. [file 12866_2020_1884_MOESM1_ESM.docx]

**Additional File 1 for**

**Diversity and distribution of marine heterotrophic bacteria from a large culture collection**

Isabel Sanz-Sáez^a^, Guillem Salazar^b^, Pablo Sánchez^a^, Elena Lara^a, c^, Marta Royo-Llonch^a^, Elisabet L. Sà^a^, Teresa Lucena^d^, María J. Pujalte^d^, Dolors Vaqué^a^, Carlos M. Duarte^e, f^, Josep M. Gasol^a^, Carlos Pedrós-Alió^g^, Olga Sánchez^h *^, Silvia G. Acinas^a*^

^a^ Department of Marine Biology and Oceanography; Institut de Ciències del Mar (CSIC); Barcelona, 08003; Spain

^b^ Department of Biology, Institute of Microbiology, ETH Zurich, Vladimir-Prelog-Weg 1-5/10, CH-8093 Zurich, Switzerland.

^c^ Institute of Marine Sciences (CNR-ISMAR), National Research Council, Castello 2737/F Arsenale-Tesa 104, 30122 Venezia, Italy

^d^ Departamento de Microbiología y Ecología and Colección Española de Cultivos Tipo (CECT), Universitat de València, Valencia, Spain

^e^ Red Sea Research Center, King Abdullah University of Science and Technology (KAUST), Thuwal 23955-6900, Saudi Arabia.

^f^ Computational Bioscience Research Center (CBRC), King Abdullah University of Science and Technology (KAUST), Thuwal 23955-6900, Saudi Arabia

^g^ Department of Systems Biology, Centro Nacional de Biotecnología (CNB), CSIC, Madrid, Spain

^h^ Departament de Genètica i Microbiologia, Facultat de Biociències, Universitat Autònoma de Barcelona, 08193 Bellaterra, Spain

^*^Correspondence: Olga Sánchez: olga.sanchez@uab.es, tel. (+34) 93 586 8022, FAX (+34) 93 581 2387; Silvia G. Acinas: [sacinas@icm.csic.es](mailto:sacinas@icm.csic.es), tel. (+34) 93 230 5886, FAX (+34) 93 230 9555

**This PDF file includes:**

Supplementary Methods

Supplementary Figures

Headings Supplementary Tables

**Supplementary Methods**

*PCR conditions*

Each PCR reaction with a final volume of 25 μl contained: 2 μl of template DNA, 0.5 μl of each deoxynucleotide triphosphate at a concentration of 10 μM, 0.75 μl of MgCl_2_ (1.5 mM final concentration), 0.5 μl of each primer reaching a final concentration of 0.5 μM, 0.125 μl (0.025u/μl) of Taq DNA polymerase (Invitrogen), 2.5 μl of PCR buffer supplied by the manufacturer (Invitrogen, Paisley, UK) and Milli-Q water up to the final volume. Reactions were carried out in a Biorad thermocycler using the following program: initial denaturation at 94ºC for 5 min, followed by 30 cycles of 1 min at 94ºC, 1 min at 55ºC and 2 min at 72ºC, and a final extension step of 10 min at 72ºC. The PCR products were verified and quantified by agarose gel electrophoresis with a standard low DNA mass ladder (Invitrogen).

*Phylogenetic trees*

Different phylogenetic trees were included in these study: (i) phylogenetic trees with all non-redundant sequences for *Alphaproteobacteria*, *Gammaproteobacteria*, *Bacteroidetes,* and Gram-positive bacteria; (ii) phylogenetic trees to support novelty of putative novel isolates (Fig. 5b in the manuscript); and (iii) one phylogenetic tree including all isolates for alpha-diversity metrics analyses (explained in the manuscript Methods).

The total pool of sequences to be included in the first two types of phylogenies were first aligned with the SINA web alignment tool (http://www.arb-silva.de/aligner/) [1] and imported into the phylogenetic software MEGA 5.2.2 [2]. The phylogenetic trees were constructed with the Neighbour Joining (NJ) algorithm using the Jukes-Cantor distance and 1000 bootstrap replicates.

*Phylogenetic trees for specific phyla or classes*

In order to see phylogenetic relationships and explore the connectivity between photic-layer, mesopelagic and bathypelagic isolates, as well as to detect possible novel strains, phylogenetic trees for *Alphaproteobacteria*, *Gammaproteobacteria*, *Bacteroidetes* and Gram positive bacteria (Supplementary Figures S2 a-d) were built. They included the non-redundant sequences dataset of each station and the taxonomic affiliation of their Closest Cultured Match (CCM) and Closest Environmental Match (CEM) obtained after BLASTn search against the RDP databases (Supplementary Tables S11 and S12 in Additional file 2).

*Samples used for Illumina 16S rRNA sequencing*

*Datasets*

Different datasets comprising Illumina 16S rRNA gene sequences were analyzed in order to compare them with 16S rRNA sequences from isolates obtained by traditional culture techniques. The first dataset comprised a total of 124 surface samples and 41 bathypelagic samples collected during the Malaspina 2010 expedition and distributed across the world’s oceans. Surface seawater (3m) samples were collected and filtered as described previously in Ruiz-González [3]. For these surface samples we focused on the 0.2–3 μm fraction, which represents mostly free‐living bacteria. On the other hand, bathypelagic samples (~4000 m depth) were collected and filtered as described in Salazar et al. [4]. In these case two different size fractions were analyzed representing the free-living (0.2-0.8 μm) and the particle-attached (0.8-20 μm) bacterial communities. The second dataset was formed by 80 surface samples and 39 mesopelagic samples collected during the *Tara* Oceans 2009 and *Tara* Oceans Polar Circle 2013 expeditions which covered the major oceanic provinces including the polar circles. Sampling strategy and methodology are described in Pesant et al. [5]. In this dataset we only focused in the free-living bacterial communities compressed within the 0.2 and 1.6 or 3 μm. In all three datasets, once seawater was processed, filters were flash-frozen in liquid nitrogen and stored at -80 ºC until DNA extraction.

*DNA extraction, amplification and sequencing*

The DNA from the samples of the different datasets described was extracted with a phenol-chloroform protocol, as described elsewhere [4, 6, 7]. Prokaryotic barcodes for each of the datasets was generated by amplifying the V4 and V5 hypervariable regions of the 16S rRNA gene using primers 515F-Y (5’-GTG YCA GCM GCC GCG GTA A-3’) and 926R (5′-CCG YCA ATT YMT TTR AGT TT-3′) described in Parada et al. [8]. Sequencing was performed in an Illumina MiSeq platform (iTAGs) using 2x250 bp paired-end approach at the Research and Testing Laboratory facility (Lubbock, TX, USA) for the Malaspina datasets and at Genoscope (Evry Cedex, France) for the *Tara* Oceans and *Tara* Polar Oceans dataset.

*16S rRNA Illumina sequences processing*

Computing analyses were run at the MARBITS bioinformatics platform at the *Institut* de *Ciències del Mar* and at the Euler scientific compute cluster of the ETH Zürich University. The obtained amplicons were processed through the bioinformatic pipeline described in the github repository <https://github.com/SushiLab/Amplicon_Recipes>. Briefly, pair-end reads were merged at a minimum 90% of identity alignment, and those with ≤ 1 expected errors were selected (quality filtering). Primer matching was performed with CUTADAPT v.1.9.1. Dereplication and zOTU (zero-radius OTUs) denoising at 100% similarity (UNOISE algorithm) were performed with USEARCH v.10.0.240 [9]. zOTUs were taxonomically annotated against the SILVA database v132 (2017) with the LCA (lowest common ancestor) approach. Finally, zOTUs were quantified to obtain zOTU-abundance tables. Non-prokaryotic zOTUs (eukaryotes, chloroplast and mitochondria) were removed, whereas singletons (zOTUs appearing only once) were maintained.

This procedure was applied individually for the: (i) 41 Malaspina bathypelagic samples, (ii) 124 Malaspina surface samples, (iii) 119 *Tara* Oceans and *Tara* Polar Oceans surface and mesopelagic samples. Hence, 3 different zOTU abundance tables were obtained after applying the pipeline. To allow comparisons between samples, each zOTU table was randomly sampled down to lowest sampling effort using the function *rrarefy.perm* with 1000 permutations from the R package *EcolUtils* (10).

*Phenotypic characterization of ISS653 and ISS1889*

Detailed phenotypic characterization is described in Lucena et al. (submitted) and here we focused on the description of those test were differences were found between strains. Briefly, it included morphological, cultural, biochemical, physiological and nutritional screening and was performed by already described methods [11]. *Mesonia algae* CECT 9441T, *Salegentibacter salegens* CECT 9443T, *Gramella echinicola* CECT 9439T and *Zunongwangia profunda* CECT 9445T were characterized in parallel for comparative purposes.

Flexirubin type pigmentation was tested according to Bernardet et al. (2002) [12]. In addition, cellulose degradation, nitrate reduction acid production from carbohydrates in API 50CH/E, APIZYM and API 20NE profile were performed as described in [13]. Fatty acid methyl esters were extracted from strains ISS653 and ISS1889 biomass grown in Marine Agar at 26 ºC after 72 h incubation. Extracts were prepared according to standard protocols as described for the MIDI Microbial Identification System [14] at the CECT. Cellular fatty acid content was analyzed by gas chromatography with an Agilent 6850 chromatographic unit, with the MIDI Microbial Identification System using the TSBA6 method [15] and identified using the Microbial Identification Sherlock software package.

**References**

1. Pruesse E, Peplies J, Glöckner FO. SINA: accurate high-throughput multiple sequence alignment of ribosomal RNA genes. Bioinformatics. 2012;28:1823–9. doi:10.1093/bioinformatics/bts252.

2. Tamura K, Peterson D, Peterson N, Stecher G, Nei M, Kumar S. MEGA5: molecular evolutionary genetics analysis using maximum likelihood, evolutionary distance, and maximum parsimony methods. Mol Biol Evol. 2011;28:2731–9. doi:10.1093/molbev/msr121.

3. Ruiz‐González C, Logares R, Sebastián M, Mestre M, Rodríguez‐Martínez R, Galí M, et al. Higher contribution of globally rare bacterial taxa reflects environmental transitions across the surface ocean. Mol Ecol. 2019;28:1930–45. doi:10.1111/mec.15026.

4. Salazar G, Cornejo-Castillo FM, Benítez-barrios V, Fraile-Nuez E, Álvarez-Salgado XA, Duarte CM, et al. Global diversity and biogeography of deep-sea pelagic prokaryotes. The ISME Journal. 2016; 10: 596–608. doi:10.1038/ismej.2015.137.

5. Pesant S, Not F, Picheral M, Kandels-Lewis S, Le Bescot N, Gorsky G, et al. Open science resources for the discovery and analysis of Tara Oceans data. Sci Data. 2015;2:150023. doi:10.1038/sdata.2015.23.

6. Massana R, Murray AE, Preston CM, Delong EF. Vertical distribution and phylogenetic characterization of marine planktonic archaea in the Santa Barbara Channel. Appl Environ Microbiol.1997;63:50-6.

7. Alberti A, Poulain J, Engelen S, Labadie K, Romac S, Ferrera I, et al. Viral to metazoan marine plankton nucleotide sequences from the Tara Oceans expedition. Sci data. 2017;4:170093. doi:10.1038/sdata.2017.93.

8. Parada AE, Needham DM, Fuhrman JA. Every base matters: Assessing small subunit rRNA primers for marine microbiomes with mock communities, time series and global field samples. Environ Microbiol. 2016;18:1403–14.

9. Edgar RC. Search and clustering orders of magnitude faster than BLAST. Bioinformatics. 2010;26:2460–1. doi:10.1093/bioinformatics/btq461.

10. Salazar, G. EcolUtils: Utilities for community ecology analysis. R package version 0.1. https://github.com/GuillemSalazar/EcolUtils. 2018.

11. Pujalte MJ, Lucena T, Rodriguez-Torres L, Arahal DR. Comparative genomics of *Thalassobius* including the description of *Thalassobius* *activus* sp. nov and *Thalassobius* *autumnalis* sp. nov. Front Microbiol 2018;8:2645.

12. Bernardet JF, Nakagawa Y, Holmes B. Proposed minimal standards for describing new taxa of the family Flavobacteriaceae and emended description of the family. Int J Syst Evol Microbiol 2002;52:1049–1070. doi: 10.1099/00207713-52-3-1049.

13. Lucena T, Arahal DR, Sanz-Saez I, Acinas SG, Sánchez O et al. *Thalassocella* *blandensis* gen. nov., sp. nov., a novel member of the family *Cellvibrionaceae*. Int J Syst Evol Microbiol 2020;70:1231–1239. doi: 10.1099/ijsem.0.003906

14. Sasser M. Identification of bacteria by gas chromatography of cellular fatty acids, MIDI Technical Note 101. 1990. Newark: DE: MIDI Inc.

15. MIDI. Sherlock Microbial Identification System Operating Manual, version 6.1. 2008. Newark, DE: MIDI Inc.


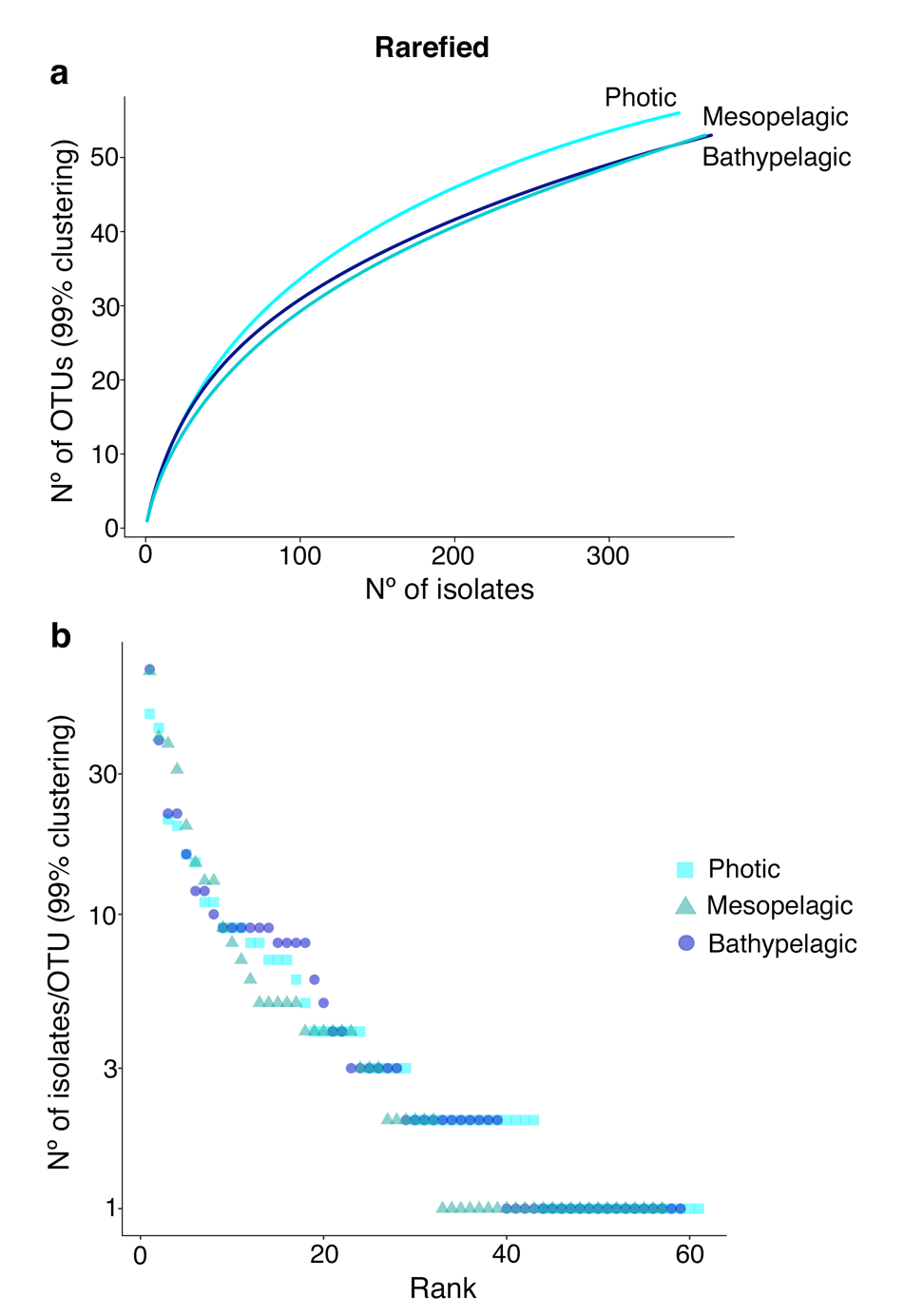
**Supplementary Figures**

**Fig. S1** **Rarefaction and rank abundance curves for each of the depths included in the study. (a)** Rarefaction curves for photic, mesopelagic, and bathypelagic samples extracted from the rarefied iOTU table down to the lowest isolated layer (mesopelagic with 362 isolates). **(b)** Rank abundance plots showing the number of isolates per OTU (at 99% clustering) obtained in the three layers studied also for the rarefied iOTU table down to the lowest isolated layer. Y axis are in log_10_ scale.

**
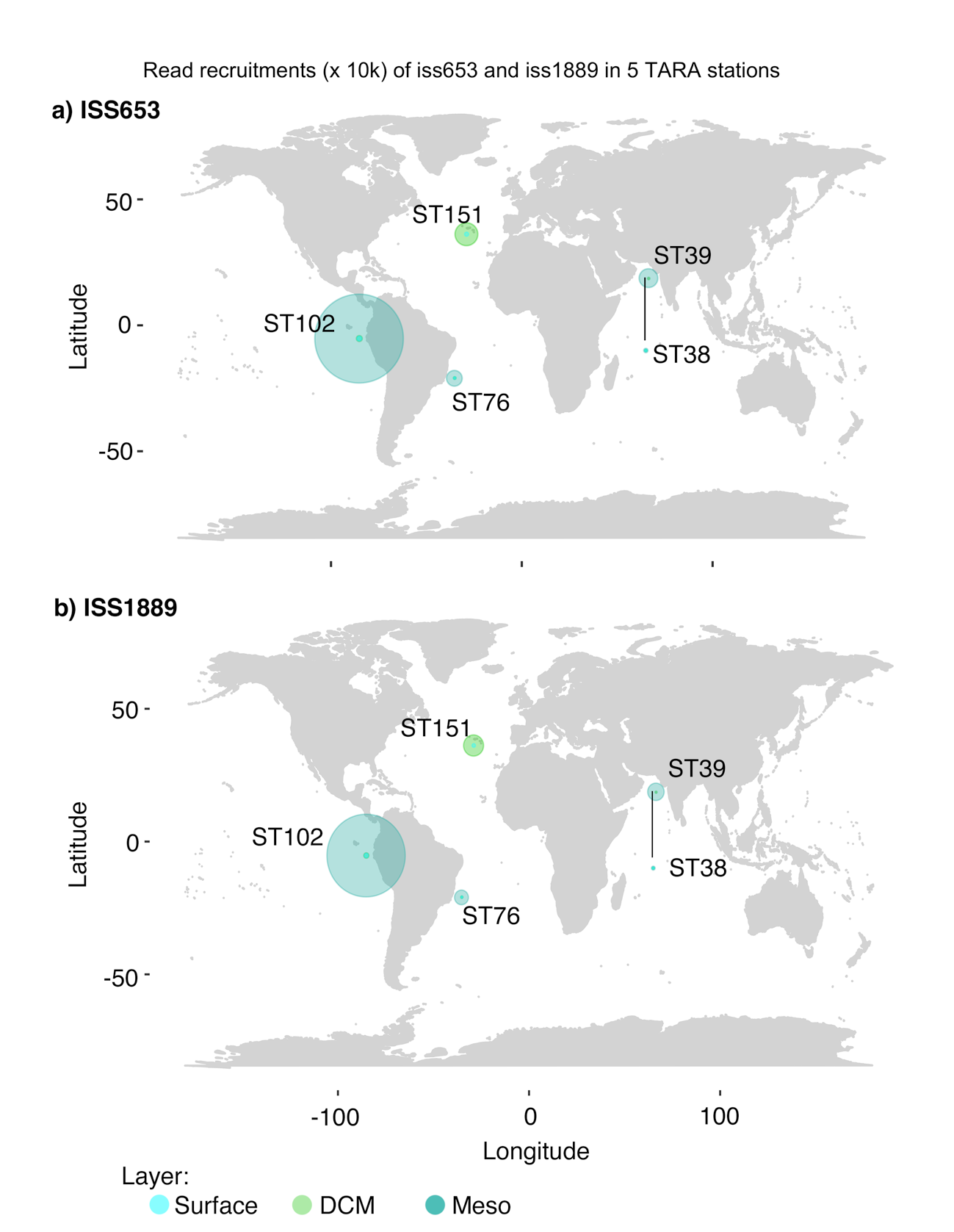
**

**Fig. S2. Map world showing the read recruitment of ISS653 and ISS1889 in five *Tara* Oceans stations.** They include the stations where the isolates were retrieved (ST151 and ST102) and some distant stations for the sake of comparison (ST39, ST38, ST76). ST38 is located near ST39 (Latitude 19º 2.24’ N, Longitude 64º 29.24’ E), but its location in the plot was slightly modified for its correct visualization. Size of the circles are the abundances of reads of each genome recruited in each station and layer (x10k). DCM, deep chlorophyll maximum; Meso, mesopelagic isolates**.**

**
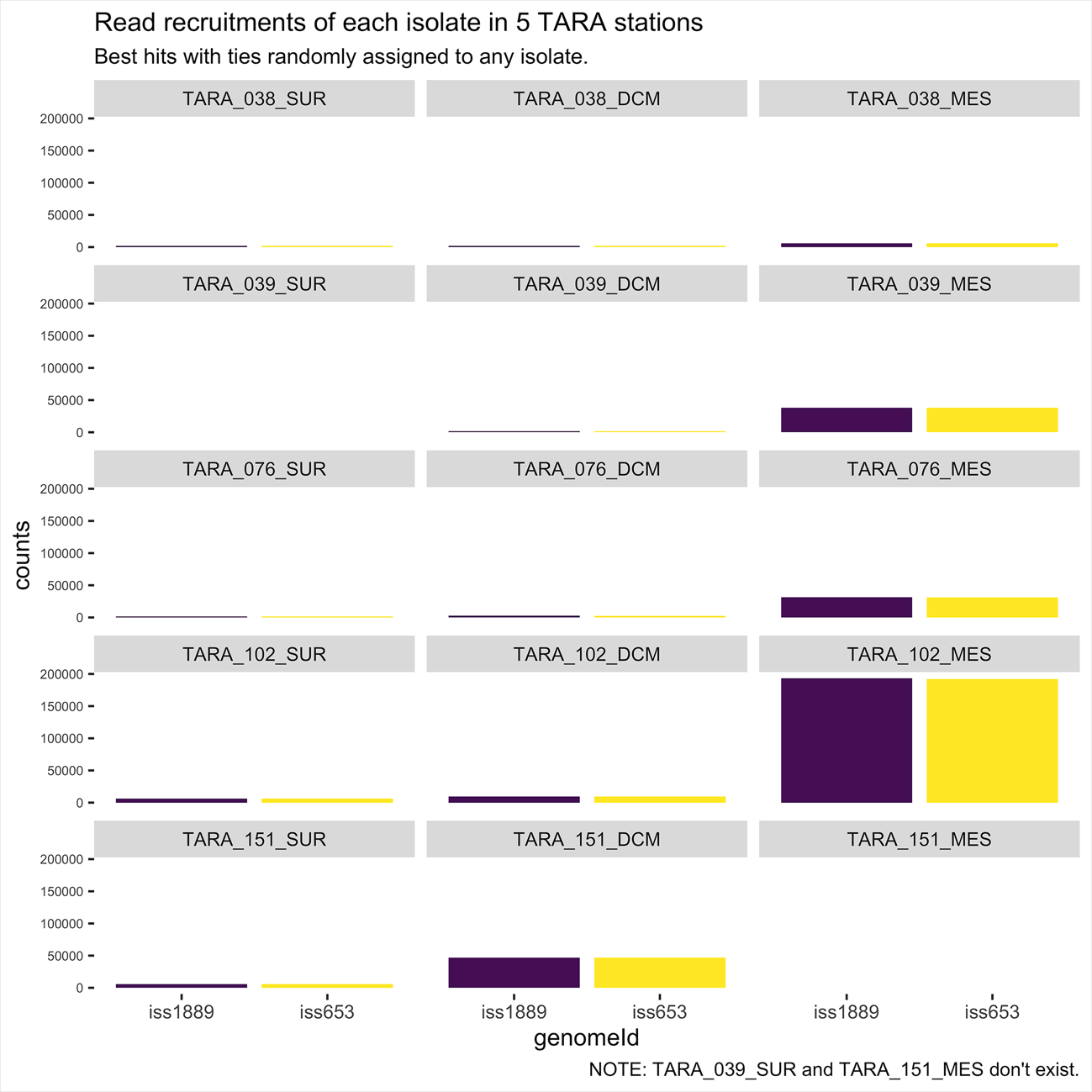
**

**Fig. S3. Read recruitment of ISS653 and ISS1889 in five *Tara* Oceans stations.** The read counts recruited in each station and layer is indicated per each genome. They include the stations where the isolates were retrieved (ST151 and ST102) and some distant stations for the sake of comparison (ST39, ST38, ST76). SUR, surface isolates; DCM, deep chlorophyll maximum; Meso, mesopelagic isolates**.**

**
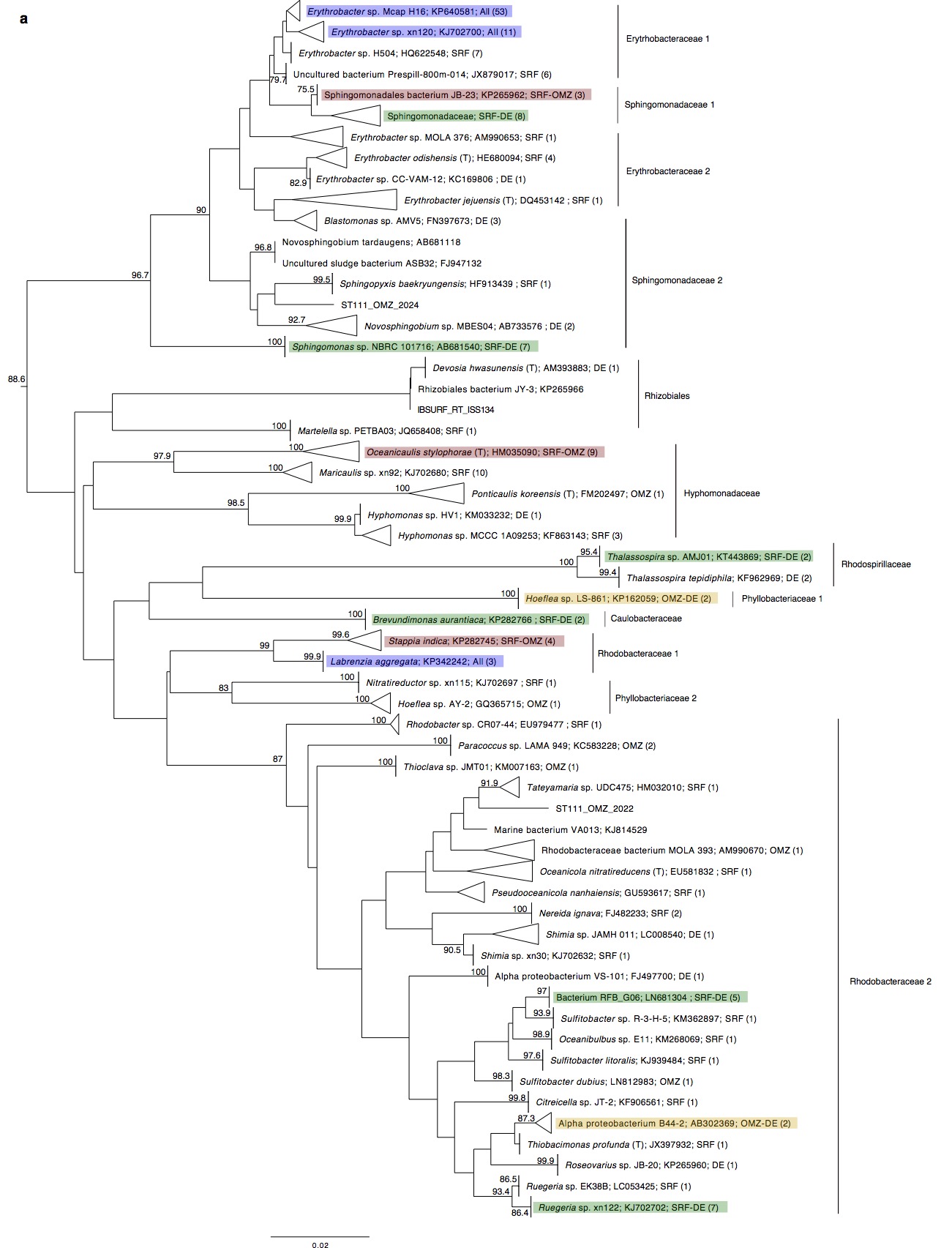
**

**
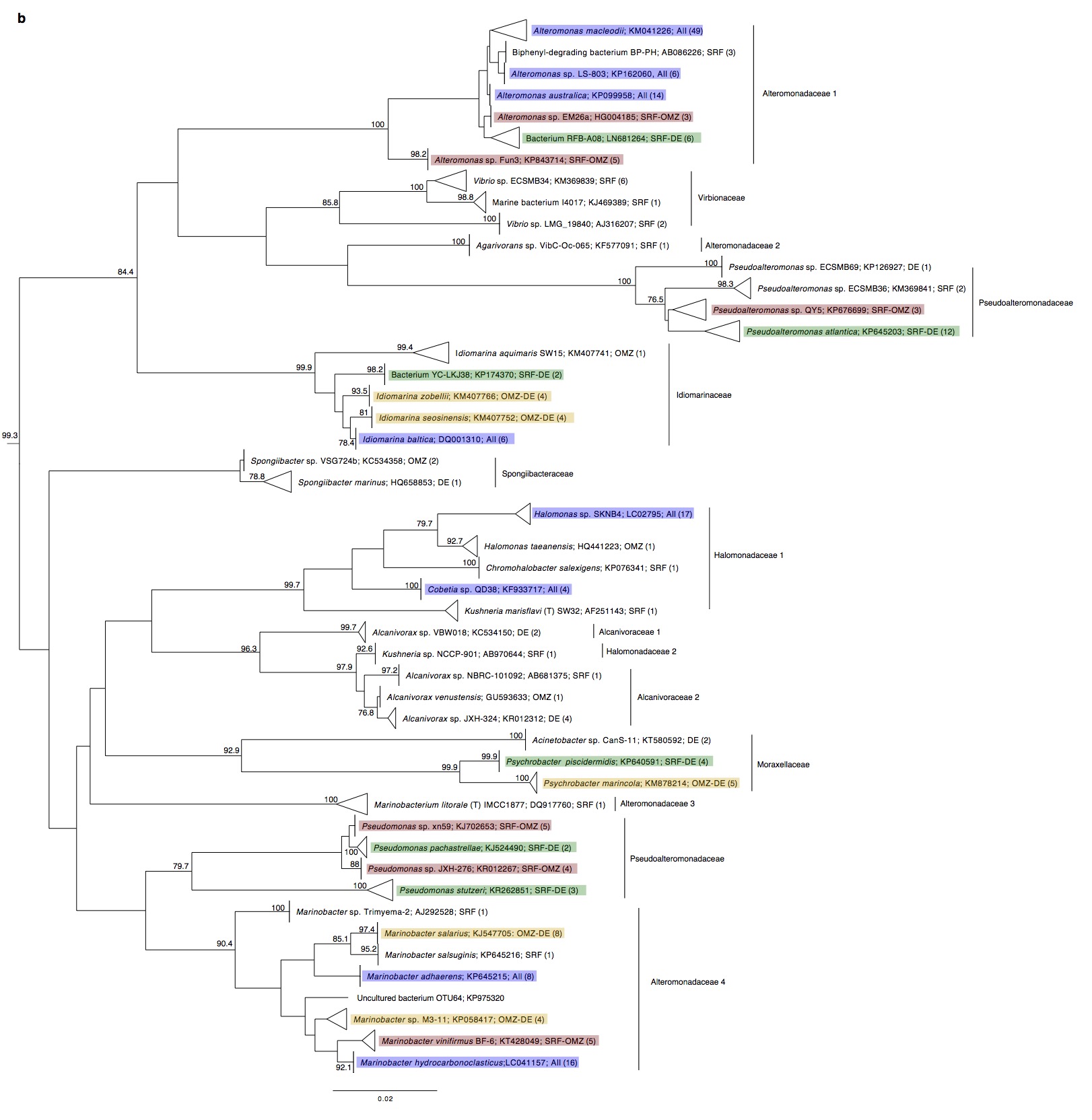
**

**
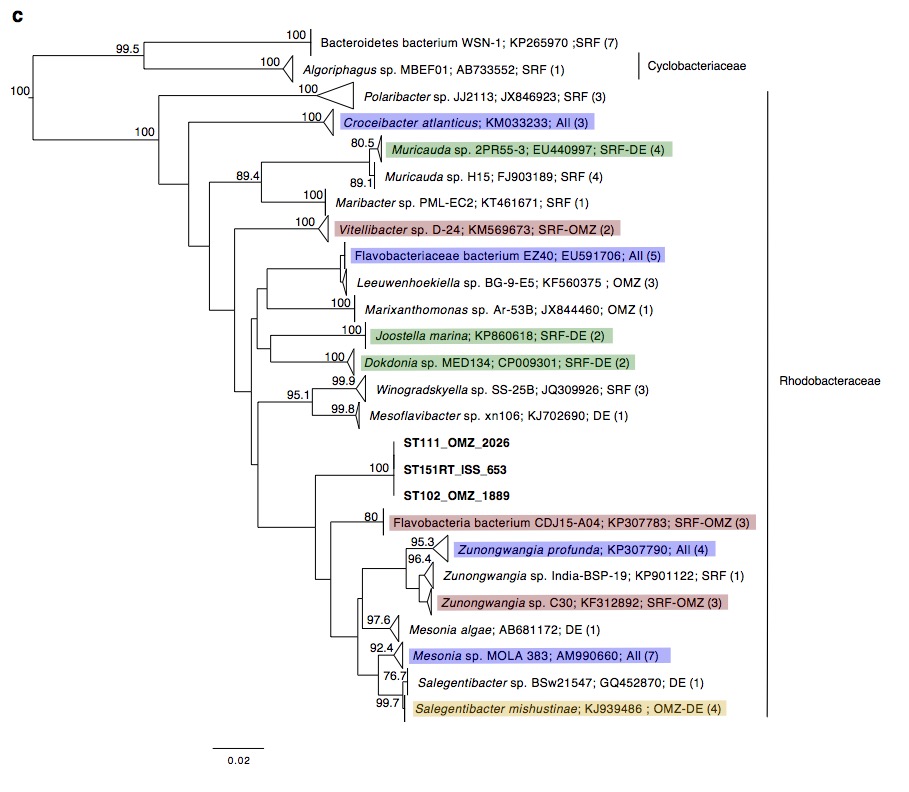
**

**
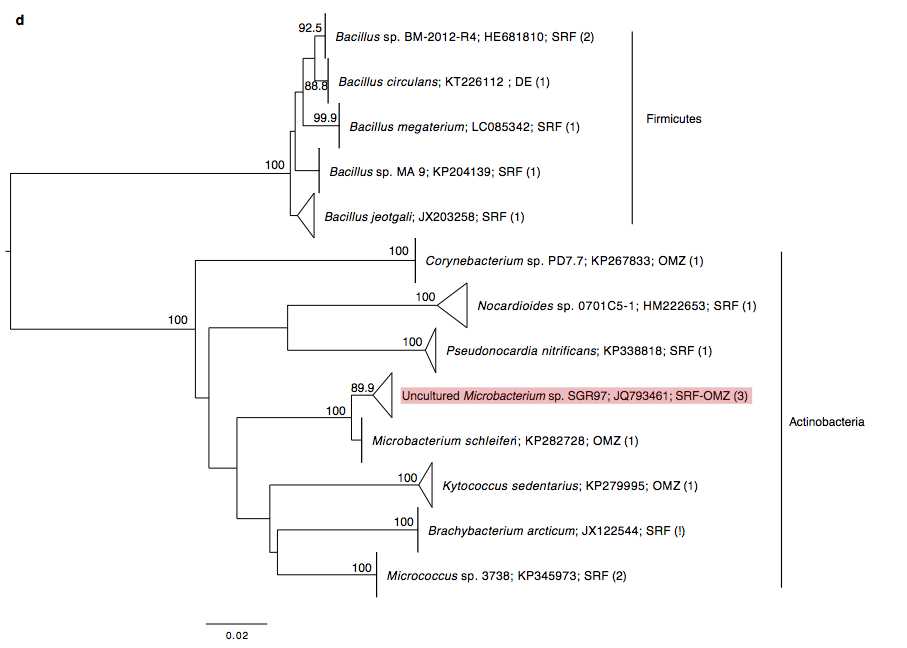
**

**Fig. S4 Phylogenetic relationships between photic-layer, mesopelagic, and bathypelagic isolates.** Neighbour Joining trees of the 16S rRNA gene sequences of the reduced pool of sequences, including the non-redundant 516 isolates plus their closest cultured match (CCM) and closest uncultured or environmental match (CEM). The numbers in nodes represents bootstrap percentages > 75, calculated from 1000 replicates. The number of isolates from each specific cluster is indicated in brackets. Isolates in bold (only in Bacteroidetes tree) show the putative novel genera isolated in this study. Blue rectangles indicate group of isolates retrieved from all depths; in red; a mix between photic-layer and mesopelagic (indicated as OMZ); in green, a mix from photic-layer and bathypelagic isolates; and in yellow, a mix of mesopelagic (indicated as OMZ) and bathypelagic. DE, bathypelagic isolates; SRF, photic-layer isolates; OMZ, oxygen minimum zone isolates which refer to the mesopelagic isolates retrieved from samples collected in areas with OMZ. The vertical lines indicate the family name or order of some groups of isolates in each tree. **(a)** *Alphaproteobacteria*; **(b)** *Gammaproteobacteria*; **(c)** *Bacteroidetes*; **(d)** Gram positive bacteria**.**

**Headings Supplementary Tables**

**Table S1.** Comparisons between the number of iOTUs (isolated OTUs) and the percentage of shared sequences between photic-layer, mesopelagic, and bathypelagic samples in non-subsampled and subsampled OTU tables.

**Table S2.** Comparisons of the richness and diversity indexes estimated using the isolates OTU tables per layer defined at 100% and 99% sequence similarity rarefied and non-rarefied to the layer with the lower number of isolates (Mesopelagic, 362 isolates). No R., stands for non-rarefied or non-subsampled OTU table, and R., stands for rarefied or subsampled OTU table. SRF, surface; MESO, mesopelagic, DEEP; bathypelagic.

**Table S3.** Subsampled iOTU-abundance table including all isolates from the photic, the mesopelagic and the bathypelagic. iOTUs obtained with clustering at 99% sequence similarity. SRF: photic-layer; DE: bathypelagic; MES: mesopelagic.

**Table S4.** Table indicating the number of total isolates affiliating to each genus found in the photic, mesopelagic, and bathypelagic samples. Results obtained after grouping all the iOTUs from the subsampled iOTU-abundance table (99% clustering) affiliating with the same genus. SRF: photic-layer isolates; MES: mesopelagic; DE: bathypelagic isolates.

**Table S5.** Table indicating the number of total isolates affiliating to each genus found in the photic, mesopelagic, and bathypelagic samples per stations. Results obtained after grouping all the iOTUs from the non-subsampled iOTU-abundance table (99% clustering) affiliating with the same genus. SRF: photic-layer isolates; MES: mesopelagic; DE: bathypelagic isolates.

**Table S6.** Non-subsampled iOTU-abundance table per depth defined at 99% sequence similarity. SRF: photic-layer; DE: bathypelagic; MES: mesopelagic.

**Table S7.** Correspondence at 100% sequence similarity between zOTUs (zero-radious OTUs) and the top12 iOTUs (99% clustering) including other iOTUs less abundant/rare matching with the same zOTU.

**Table S8.** New potential isolates best hits. Closest Cultured Match (CCM) and Closest Environmental/Uncultured Match (CEM) of the ISS653, ISS1889 and ISS2026 isolates BLASTn results against the NCBI, RDP 11 and SILVA LTP databases. Accession number and percentage of similarity are indicated together with the best hit.

**Table S9.** Annotated proteins detected in only one of the genomes of the new *Mesonia* strains. 33 proteins are codified only in ISS653, whereas 6 are unique in ISS1889.

**Table S10.** Culture media and incubation conditions used for each seawater sample. Positive signs indicate which media where used. RT: room temperature.

**Table S11.** Metadata information of the Closest Cultured Match (CCM) obtained after BLASTn analysis of the isolates against a subset of the RDP database including only sequences from previously published cultured bacteria. SRF: photic-layer; DE: bathypelagic; MES: mesopelagic.

**Table S12**. Metadata information of the Closest Environmental Match (CEM) obtained after BLASTn analysis of the isolates against a subset of the RDP database including only sequences from previously published uncultured bacteria. SRF: photic-layer; DE: bathypelagic; MES: mesopelagic.
